# Plasmids and Rickettsial Evolution: Insight from *Rickettsia felis*

**DOI:** 10.1371/journal.pone.0000266

**Published:** 2007-03-07

**Authors:** Joseph J. Gillespie, Magda S. Beier, M. Sayeedur Rahman, Nicole C. Ammerman, Joshua M. Shallom, Anjan Purkayastha, Bruno S. Sobral, Abdu F. Azad

**Affiliations:** 1 Virginia Bioinformatics Institute at Virginia Tech, Blacksburg, Virginia, United States of America; 2 Department of Microbiology and Immunology, University of Maryland School of Medicine, Baltimore, Maryland, United States of America; Utrecht University, Netherlands

## Abstract

**Background:**

The genome sequence of *Rickettsia felis* revealed a number of rickettsial genetic anomalies that likely contribute not only to a large genome size relative to other rickettsiae, but also to phenotypic oddities that have confounded the categorization of *R. felis* as either typhus group (TG) or spotted fever group (SFG) rickettsiae. Most intriguing was the first report from rickettsiae of a conjugative plasmid (pRF) that contains 68 putative open reading frames, several of which are predicted to encode proteins with high similarity to conjugative machinery in other plasmid-containing bacteria.

**Methodology/Principal Findings:**

Using phylogeny estimation, we determined the mode of inheritance of pRF genes relative to conserved rickettsial chromosomal genes. Phylogenies of chromosomal genes were in agreement with other published rickettsial trees. However, phylogenies including pRF genes yielded different topologies and suggest a close relationship between pRF and ancestral group (AG) rickettsiae, including the recently completed genome of *R. bellii* str. RML369-C. This relatedness is further supported by the distribution of pRF genes across other rickettsiae, as 10 pRF genes (or inactive derivatives) also occur in AG (but not SFG) rickettsiae, with five of these genes characteristic of typical plasmids. Detailed characterization of pRF genes resulted in two novel findings: the identification of *oriV* and replication termination regions, and the likelihood that a second proposed plasmid, pRFδ, is an artifact of the original genome assembly.

**Conclusion/Significance:**

Altogether, we propose a new rickettsial classification scheme with the addition of a fourth lineage, transitional group (TRG) rickettsiae, that is unique from TG and SFG rickettsiae and harbors genes from possible exchanges with AG rickettsiae via conjugation. We offer insight into the evolution of a plastic plasmid system in rickettsiae, including the role plasmids may have played in the acquirement of virulence traits in pathogenic strains, and the likely origin of plasmids within the rickettsial tree.

## Introduction

All members of the genus *Rickettsia* (Rickettsiales; Rickettsiaceae) are obligate intracellular parasites of eukaryotes [1], with some species that are pathogenic and are known to cause harmful diseases in humans, e.g., *R. prowazekii*, the causative agent of epidemic typhus and *R. rickettsii*, the agent of Rocky Mountain spotted fever [2]. Some rickettsiae are important both as emerging pathogens [3] and as selected agents for the development of bioweapons [4]. Given the diversity of *Rickettsia* spp. [5], [6] and their associated pathologies, a sound understanding of the evolutionary relationships of these diverse bacteria is essential for species-level diagnostics and potential vaccine targeting. The system of classification of rickettsiae has traditionally grouped *Rickettsia* spp. into the spotted fever (SFG) and typhus group (TG) rickettsiae [2] with *R. bellii* considered ancestral to both groups [7], [8]. *R. canadensis* was subsequently added to this ancestral group (AG) rickettsiae [9]. Recent phylogenies based on various molecular markers have resulted in conflicting tree topologies [e.g., 10,11,12,13]; however, when AG rickettsiae are included they are nearly always basal to the remaining TG and SFG rickettsiae clades [1], [14], [15]. Moreover, only with the inclusion of AG rickettsiae (for rooting the analyses) is a unique lineage recovered consisting of *R. akari* and *R. felis* [1], [14], [15], as well as *R. australis* and some other unidentified rickettsiae from booklouse (*Liposcelis* sp.) and parasitic wasp (*Neochrysocharis* sp.) hosts [1]. This clade, referred to hereafter as transitional group (TRG) rickettsiae, is interesting from a host perspective because, while both *R. akari* and *R. felis* have been classified as SFG rickettsiae, neither species purportedly parasitizes a tick host, with *R. akari* found in mites [16] and *R. felis* found in fleas [17], [18], [19], [20], [21].

*R. felis* has been difficult to place phylogenetically because it displays some genotypic and phenotypic attributes of both SFG and TG rickettsiae, e.g., association with insect, hemolytic activity, actin-based motility, transovarial maintenance in the vector hosts, and serological cross-reactivity. In addition, the genome sequence of *R. felis* revealed morphological surprises, such as the presence of plasmids (pRF) and conjugative pili, as well as genetic traits atypical of most rickettsial genomes, such as elevated copies of several transposase families, *spoT* genes and genes coding for uncharacterized proteins with ankyrin (ANK) and tetratricopeptide (TPR) motifs [21], [22]. Despite tremendous laboratory efforts these features had not been identified in *Rickettsia* in the pre-genomic era [23].

The discovery of a plasmid system in *R. felis* is rather peculiar since no other bacteria in the Rickettsiales (*Ehrlichia, Anaplasma, Neorickettsia*, and *Wolbachia*) are known to harbor plasmids based on their available completed genome sequences. Plasmids are known only from a few other obligate intracellular bacteria, including the γ-proteobacterium Q fever agent *Coxiella burnetti* [24], [25], str. MCS of an unclassified species of *Mycobacterium* [26], and the distantly related Chlamydiaceae species *Chlamydia trachomatis* [27], [28], [29], *Chlamydia muridarum* [30], *Chlamydophila pneumoniae* [30], [31], *Chlamydophila psittaci* [32], [33], [34], *Chlamydophila caviae* [35], and *Chlamydophila felis* [36]. The existence of a putative conjugative plasmid presents a historical riddle when *R. felis* is considered within a phylogenetic context. *R. felis* is neither ancestral nor derived within the rickettsial evolutionary tree [1], [14], [15], yet it is the only published rickettsial genome to date that contains autonomous and seemingly functional plasmids [21], [22]. This entails one of two evolutionary scenarios for rickettsiae. First, all ancestral rickettsiae once contained functional plasmids that have been lost in all lineages for which a genome has been sequenced (except *R. felis*). Given the phylogenetic position of *R. felis*, this implies multiple losses of the plasmid and raises the question of why, among all ten sequenced rickettsial genomes, a single maintenance of a plasmid system would remain in *R. felis*. Under this hypothesis, if some plasmid genes are essential for rickettsial fitness, then the lineages without plasmids may have had the plasmid genes incorporated into their chromosomes where they have become a permanent fixture, as is the case for the plasmidless *C. burnetti* isolate Scurry Q217 [37], [38] and some plasmidless *Chlamydia* spp. [35], [39], [40]. Thus, pRF genes that are not present in other rickettsiae would likely be involved in the specific biology of *R. felis*, and may involve critical proteins for its survival and/or virulence. Otherwise they could be pseudogenes in the early stages of decay.

Alternatively, perhaps *R. felis* acquired a plasmid from another organism and has retained this plasmid through the incorporation of certain genes on the plasmid into its life cycle such that loss of these genes would be deleterious for the survival and/or virulence of *R. felis*. This hypothesis implies that pRF genes would be less related to other rickettsial orthologous genes than are genes on the *R. felis* chromosome. Garnering support for either evolutionary model requires the evaluation of each plasmid gene through phylogenetic estimation and comparative analysis with other organisms that may likely have contributed to the structure and composition of pRF via vertical (phylogenetic) and horizontal (e.g., conjugative) gene transfer (HGT).

Ogata et al. [22] proposed that the genes found on the plasmid and chromosome of *R. felis* are homologous (sharing common ancestry) and likely undergo exchanges between the replicons. To test this claim, as well as to rule out one of the two hypotheses stated above, we present a phylogenetic analysis that discerns the mode of inheritance of the genes on the pRF plasmid that are also found on the chromosome of *R. felis* and other rickettsiae. Furthermore, we characterize the composition of the pRF plasmid in light of other plasmid-containing obligate intracellular bacteria, adding novel information that strengthens the hypothesis that pRF is conjugative and self-replicating. This comparison of the evolution of independent replicons in *R. felis* will ultimately lend resolution to the ambiguity that has long plagued the systematic placement of this perplexing rickettsial taxon.

## Results and Discussion

### Rickettsiae Phylogenomics

**Figure 1:**
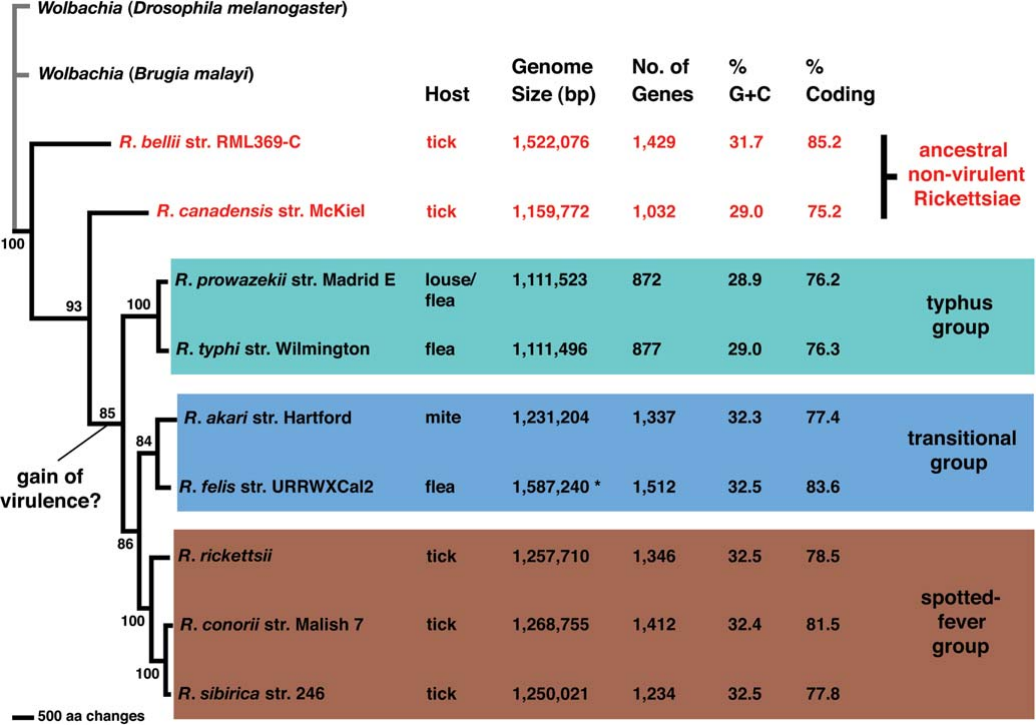
Phylogeny estimation from analysis of fifteen *R. felis* proteins. Phylogeny estimation under parsimony of fifteen *R. felis* proteins (hypothetical protein RF_0005, threonyl-tRNA synthetase, preprotein translocase SecA subunit, uncharacterized low-complexity protein RF_0864, pyruvate phosphate dikinase precursor, leucyl-tRNA synthetase, hypothetical protein RF_0556, NAD-specific glutamate dehydrogenase, DNA polymerase III alpha chain, O-antigen export system permease protein RfbA, thioredoxin, NADPH-dependent glutamate synthase beta chain and related oxidoreductases, putative TIM-barrel protein in nifR3 family, UDP-3-O-[3-hydroxymyristoyl] glucosamine, and zinc/manganese ABC transporter substrate binding protein TroA_c) from nine rickettsial species (*Rickettsia bellii, R. canadensis, R. prowazekii, R. typhi, R. akari, R. felis, R. conorii, R. rickettsii*, and *R. sibirica*) and two strains of *Wolbachia*. Branch support is from one million bootstrap replicates. Genome information was compiled from the PATRIC Website. * Total *R. felis* genome size: 1,485,148 bp = chromosome; 62,829 bp = pRF and 39,263 bp = pRFδ.

An estimated phylogeny from 15 chromosomal-encoded proteins present in nine rickettsial genomes and two strains of *Wolbachia* endosymbionts, is presented in Figure 1 and used as a reference for the robust relationships of the four main lineages of rickettsiae (AG, TG, TRG, SFG). This single most parsimonious tree, 8061 steps in length, was the result of an exhaustive search that analyzed all possible trees from treespace. From a total of the 12263 amino acid characters in the concatenated alignment, 3039 were parsimony-informative. This phylogeny is congruent with other recently published rickettsial molecular phylogenies [1], [14], [15], thus supporting our establishment of the TRG rickettsiae as a lineage distinct from other previously established taxonomic categories for rickettsiae.

Summary information of nine published rickettsial genomes illustrates the increased genome size of *R. felis* relative to other non-AG rickettsiae (Figure 1). While *R. felis* genome size is most similar to *R. bellii* str. RML369-C, it is interesting that the presence of pRF does not result in a remarkably larger genome size, given the lack of plasmids in *R. bellii* str. RML369-C [15]. In light of the recent discovery of pili in *R. bellii* str. RML369-C [15], it may be possible that genes once present on a plasmid have been incorporated into the chromosome, accounting for the larger size of the *R. bellii* genome relative to other rickettsiae (save *R. felis*). Other *R. felis* genome summary statistics, including gene number, percent GC and percent coding, are not significantly different than other rickettsial genomes (Figure 1).

### Phylogenies of Chromosomal and pRF genes

**Figure 2:**
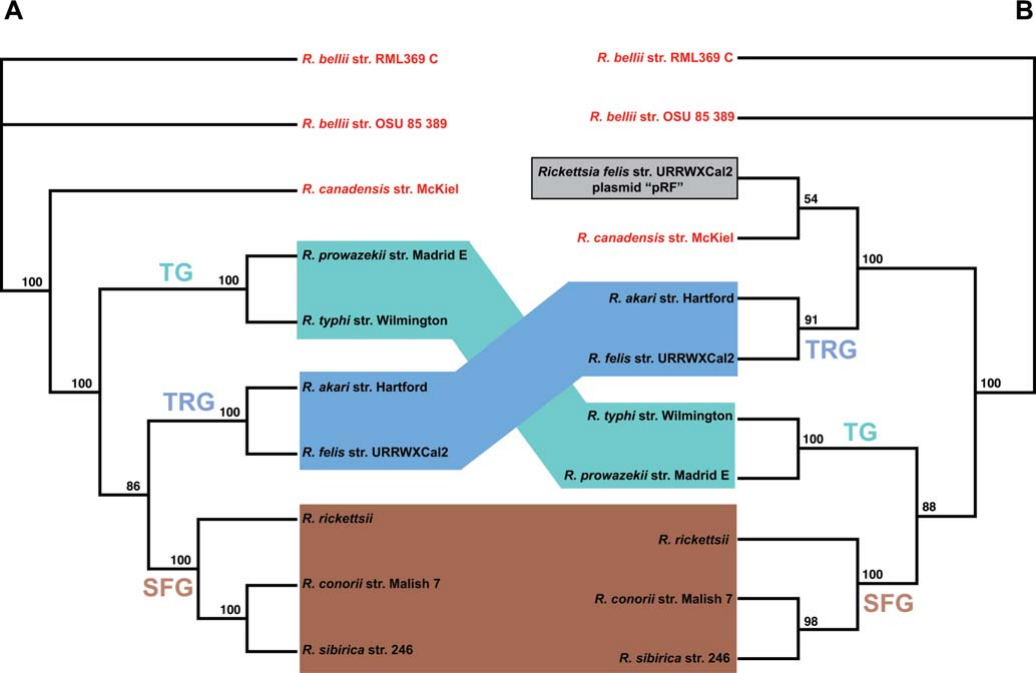
Comparison of phylogeny estimations from exclusively chromosomal proteins and proteins present on the chromosome and plasmids of *R. felis.* (A) Estimated phylogeny of 21 exclusively chromosomal proteins from 10 rickettsial strains. (B) Estimated phylogeny of 10 proteins present on the chromosome and plasmids of *R. felis*. “Ancestral” (red) refers to primitive rickettsiae with no known potential for host virulence. TG (aquamarine) = typhus group, TRG (light blue) = transitional group and SFG (brown) = spotted fever group. TG and TRG boxes depict the major differences in tree topologies. The pRF genes are boxed and shaded. Results from both analyses of amino acids are from an exhaustive search under parsimony with branch support from one million bootstrap replications.

A phylogeny estimated from 21 conserved hypothetical proteins found only on the chromosomes of 10 *Rickettsia* spp. (Figure 2A) is similar to our phylogeny based on six fewer genes (Figure 1) as well as other recently published rickettsial trees [1], [14], [15], suggesting that the conserved rickettsial hypothetical proteins are phylogenetically informative markers. This suggests that these uncharacterized ORFs code for functional proteins, as we might expect less conserved genes or pseudogenes to elicit a phylogenetic signal different from the organismal phylogeny [41], [42], [43]. Even though these 21 proteins are hypothetical, the bootstrap values increased for several of the branches compared to the tree based on 15 proteins (Figure 1), suggesting that adding more data for phylogeny estimation is better for recovering the apparent relatedness amongst these 10 *Rickettsia* spp., an approach recently verified using concatenated nucleotide alignments [14]. Thus, the failure for this phylogenetic position of *R. felis* to be recovered in many previous studies is likely due to the fewer number of genes analyzed. For instance, when we analyzed nine genes, we recovered the same tree topology but with weaker bootstrap support (data not shown). Furthermore, analyses of fewer than nine genes did not consistently recover the *R. akari*/*R. felis* clade. In light of these findings, we strongly caution against the use of single or few genes to estimate historical divergence within rickettsiae. This problem can be seen in the comparison of recent trees estimated from 16S rDNA sequences. While the tree from Perlman et al. [1] is in general agreement with our estimates (Figure 1 & 2A) and that of Abergel et al. [14], the 16S rDNA trees from Lawson et al. [44], Kikuchi et al. [12], Fournier et al. [6], and our unpublished analyses do not recover the AG rickettsiae as basal to the remaining *Rickettsia* spp. that are pathogenic in vertebrates. These conflicting trees are likely due less to differences in analytical methods than variance in the sampled taxa, thus highlighting that taxon sampling is just as important as concatenating multiple datasets for estimating a robust phylogeny of rickettsiae.

**Figure 3:**
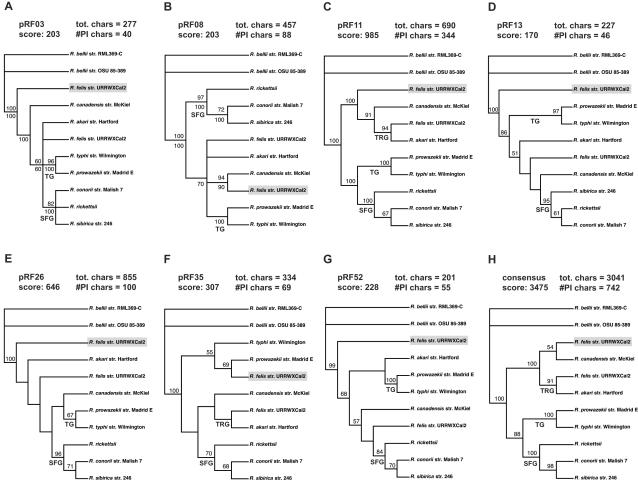
Individual phylogeny estimations for the seven pRF proteins used in the combined analysis of pRF. (A, B) Majority rule consensus trees. (C–H) Strict consensus trees. All analyses were of amino acids from an exhaustive search under parsimony with branch support from one million bootstrap replications. Bootstrap values are placed above branches. Percentages of nodes recovered in majority rule consensus trees are shown below branches. Scores are tree lengths, with total characters and number of parsimony informative characters provided.

While our generated phylogenies described above (Figure 1, 2) are robust and in agreement with other well-supported studies, the consensus phylogeny of the seven ORFs found on both pRF and the *R. felis* chromosome, as well the chromosomes of the other sampled rickettsiae, tells a different story (Figure 2B). Most unexpected is the non-monophyly of the pRF and chromosomal genes of *R. felis*, which strongly suggests that the analyzed pRF genes were not vertically passed over time in the lineage leading to *R. felis*, but rather were likely inherited horizontally from other bacterial plasmids and non-bacterial DNA. Furthermore, none of the individual plasmid gene trees are in agreement with the chromosomal phylogeny (Figure 3). The majority of the single trees place the plasmid genes basal to the non-*R. bellii* taxa (Figure 3A, C, D, E, G). One tree groups the plasmid genes closely with *R. canadensis* (Figure 3B), while one tree is mostly discordant with any reasonable rickettsial phylogeny (Figure 3F). Thus, both consensus and individual pRF phylogenies suggest an affinity of pRF with AG rickettsiae over any other rickettsial group. The inclusion of the pRF genes in rickettsial phylogeny estimation has a profound impact on tree topology, as TG and SFG rickettsiae are grouped as monophyletic to the exclusion of TRG rickettsiae, which groups with *R. canadensis* and pRF (Figure 2B, 3H). This is perhaps our most compelling evidence for the separation of TRG rickettsiae from SFG rickettsiae.

Our observation that pRF genes are closely related to AG rickettsial genes is further supported by analyzing the distribution of the *R. felis* genes found exclusively on pRF (Table 1). Thirty-eight of the 68 pRF genes are not found on the *R. felis* chromosome, and none of these 38 genes are present in any of the SFG. Furthermore, only three exclusively pRF genes occur in TG and one truncated gene is found in *R. akari*. However, 10 exclusively pRF genes (or inactive derivatives) occur in the ancestral rickettsiae, with five of these genes characteristic of typical plasmids. While our phylogenetic analysis suggests that the pRF plasmid of *R. felis* is likely composed of many products of HGT, possibly through conjugation with other bacteria (especially AG rickettsiae), the question still remains: *of all sequenced rickettsial genomes, why do plasmids occur exclusively in R. felis?* We attempt to answer this in light of the characterization of the plasmid genes and their distribution in *R. felis* chromosome, other rickettsiae, other bacterial taxa, and other non-bacterial organisms.

**Table 1:** Annotation of *R. felis* large (pRF) and small (pRFδ) plasmid-encoded genes that are absent on the *R. felis* chromosome, and their distribution in other rickettsiae, other bacterial sequences, and other non-bacterial taxa.

pRF and pRFδ (26)				Distribution b												
ORF a	Name	Annotation	%GC	AG			TG		TRG		SFG			bac	nb	pl
				Br	Bo	Ca	Pr	Ty	Ak	Fe	Ri	Co	Si	y/n/f	y/n	y/n
**pRF04**	—	*R. felis* specific protein	0.24	—	—	—	—	—	—	2	—	—	—	y	y	n
pRF05	—	chromosomal replication initiator protein DnaA-like protein	0.32	1	1	—	—	—	—	4	—	—	—	f	y	n
pRF07	*HsdR*	type I restriction-modification system methyltransferase subunit	0.32	r2	r2	r	—	—	t	2	—	—	—	y	n	n
**pRF09**	—	*R. felis* specific protein (not found in other life)	0.35	—	—	—	—	—	—	2	—	—	—	n	n	n
**pRF12**	*tpr*	tetratricopeptide repeat domain (TPR)	0.34	—	—	—	—	—	—	3,t	—	—	—	f	y	n
pRF14	*ank*	ankyrin-repeat containing gene (ANK)	0.39	—	—	—	r	—	—	2	—	—	—	f	y	n
pRF39	—	MobA_MobL (plasmid transfer)/RecD (exonuclease V) hybrid	0.38	s	s	—	—	—	—	2	—	—	—	y	y	n
**pRF40**	—	*R. felis* specific protein	0.35	—	—	—	—	—	—	2	—	—	—	f	y	n
pRF44	*traDF*	putative conjugative transfer protein TraD (*E. coli* F plasmid)	0.37	1	1	t	—	—	—	2	—	—	—	y	n	n
**pRF45**	—	*R. felis* specific protein	0.28	—	—	—	—	—	—	2	—	—	—	f	y	n
pRF46	*traGF*	putative conjugative transfer protein TraG (*E. coli* F plasmid)	0.36	1	1	—	—	—	—	2	—	—	—	f	y	n
pRF47	*traGF*	putative conjugative transfer protein TraG (*E. coli* F plasmid)	0.40	1	1	t	—	—	—	2	—	—	—	y	n	n
pRF48	*rve*	integrase (integration of viral DNA into the host chromosome)	0.35	—	—	t	—	—	—	2	—	—	—	y	n	n
pRF49	—	similar to integrase	0.30	—	—	t	—	—	—	2	—	—	—	y	n	n
**pRF50**	—	hypothetical protein conserved in a few other bacteria	0.35	—	—	—	—	—	—	2	—	—	—	f	n	n
**pRF53**	—	DNA polymerase III, epsilon subunit-like protein	0.33	—	—	—	—	—	—	2	—	—	—	y	n	y
**pRF56**	—	hyaluronidase (increases tissue permeability/antigenic disguise)	0.31	—	—	—	—	—	—	2	—	—	—	y	y	y
**pRF57**	*trp_20*	transposase 20: IS116/IS110/IS902 family [pfam02371]	0.33	—	—	—	—	—	—	2	—	—	—	y	n	y
**pRF58**	*trp*	COG3547: transposase and inactivated derivatives	0.34	—	—	—	—	—	—	2	—	—	—	y	n	y
**pRF59**	—	*R. felis* specific protein (not found in other life)	0.32	—	—	—	—	—	—	2	—	—	—	n	n	n
**pRF60**	—	similar to IS element transposase (*E. coli*)	0.32	—	—	—	—	—	—	2	—	—	—	y	n	n
**pRF62**	—	*R. felis* specific protein; possible tldD/PmbA protein	0.35	—	—	—	—	—	—	2	—	—	—	f	y	n
**pRF63**	—	*R. felis* specific protein; similar to *Wolbachia* repA	0.38	—	—	—	—	—	—	2	—	—	—	y	y	y
**pRF66**	—	site-specific recombinases (DNA invertase Pin homologs)	0.34	—	—	—	—	—	—	2	—	—	—	y	n	y
**pRF67**	—	similar to transposase ISSag8 (*Streptococcus agalactiae* A909)	0.27	—	—	—	—	—	—	2	—	—	—	y	n	n
pRF68	—	rickettsial hypothetical protein	0.34	1	s	—	1	1	—	2	—	—	—	f	n	n
**pRF (12)**				**Distribution** b												
**ORF** a	**Name**	**Annotation**	**%GC**	**AG**			**TG**		**TRG**		**SFG**			**bac**	**nb**	**pl**
				**Br**	**Bo**	**Ca**	**Pr**	**Ty**	**Ak**	**Fe**	**Ri**	**Co**	**Si**	**y/n/f**	**y/n**	**y/n**
**pRF15**	*tpr*	tetratricopeptide repeat domain (TPR)	0.36	—	—	—	—	—	—	1	—	—	—	f	y	n
pRF20	—	chromosomal replication initiator protein DnaA-like protein	0.29	—	—	—	—	—	—	1	—	—	—	f	y	n
**pRF21**	—	*R. felis* specific protein; possible transcription repressor protein	0.31	—	—	—	—	—	—	1	—	—	—	f	n	n
**pRF22**	—	similar to *P. syringae* plasmid Ppsr1 ORF12	0.32	—	—	—	—	—	—	1	—	—	—	f	y	y
**pRF24**	*tpr*	tetratricopeptide repeat domain (TPR); similar to *sca12*	0.40	—	—	—	—	—	—	2	—	—	—	f	n	n
pRF28	—	rickettsial hypothetical protein	0.33	—	—	—	1	1	—	1	—	—	—	f	y	n
**pRF32**	*tnpR*	TnpR resolvase (plasmid-encoded site-specific recombinase)	0.38	—	—	—	—	—	—	1	—	—	—	y	n	y
**pRF33**	—	*R*. *felis* specific protein	0.36	—	—	—	—	—	—	1	—	—	—	f	y	n
**pRF34**	—	DNA polymerase III, epsilon subunit-like protein; WGR domain	0.32	—	—	—	—	—	—	1	—	—	—	y	n	y
**pRF36**	—	*R. felis* specific protein	0.28	—	—	—	—	—	—	1	—	—	—	n	y	n
pRF37	—	conjugative transfer protein TraD Ti (*A. tumefaciens* Ti plasmid)	0.34	1	1	r	—	—	—	1	—	—	—	y	n	y
**pRF38**	—	conjugative transfer protein TraA Ti (*A. tumefaciens* Ti plasmid)	0.38	—	—	—	—	—	—	1	—	—	—	y	y	y

a ORF labels that are bolded depict putative genes that are unknown from other published rickettsiae genomes.

b AG = ancestral group, TG = typhus group, TRG = transitional group, SFG = spotted fever group, bac = present (y) or absent (n) in a diverse array of non-rickettsial bacteria, or present in a few (f) bacteria, nb = present (y) or absent (n) in non-bacterial organisms. Presence (y) or absence (n) of putative orthologs found in other plasmids (pl) are listed. Br = *R. bellii* str. RML369-C, Bo = *R. bellii* str. OSU 85 389, Ca = *R. canadensis* str. McKiel, Pr = *R. prowazekii* str. Madrid E, Ty = *R. typhi* str. Wilmington, Ak = *R. akari* str. Hartford, Fe = *R. felis* str. URRWXCal2, Ri = *R. rickettsii*, Co = *R. conorii* str. Malish 7, and Si = *R. sibirica* str. 246. r = reduced gene relative to the plasmid gene, t = truncated gene relative to the plasmid gene, s = split gene relative to the plasmid gene.

### Characterization and Distribution of pRF Genes

The pRF plasmid in *R. felis* could be maintained for a variety of reasons, all of which are not mutually exclusive. First, since it contains proteins with high homology to bacterial conjugation proteins (*tra* genes), pRF could function in fertility as a *(F)plasmid*, exchanging genetic material with other bacterial congeners via a pilus. Support for this role comes from the presence of putative DNA transfer proteins TraA Ti (pRF38/39), TraD Ti (pRF37) and TraD (pRF43/44), and two F-pilus assembly/aggregate stabilization homologs of *E. coli* TraGF (pRF46 and pRF47). Other important conjugative proteins are found on the *R. felis* chromosome, including two competence protein ComE3 orthologs (RF0020 and RF0021), a DNA primase (RF0786) similar to the *E. coli* TraC protein that replicates transferred DNA in recipient cells, a protein (RF0705/RF0706) similar to the P-pilus assembly protein FimD, and a protein (RF0964) similar to *E. coli* F-pilin acetylation protein TraX [22]. Thus, the majority of conjugative transfer genes and other structural proteins involved in conjugation are present in the *R. felis* genome, suggesting at the very least that *R. felis* once had the capacity to transfer plasmid genes. This is further supported by the presence of some of these proteins in other rickettsiae (Table 1 and 2), particularly the ancestral taxa. As previously noted [22], the presence of conserved and fully intact type IV secretion system (T4SS) components (found in all rickettsiae) could allow for efficient transfer of plasmid DNA, much like that found in the *vir* and *dot/icm* T4SSs of *Agrobacterium tumefaciens* and *Legionella pneumophila*, respectively, that have dual functions as DNA-transfer machines and effector protein translocators [45].

**Table 2:** Annotation of *R. felis* large (pRF) and small (pRFδ) plasmid-encoded genes that are present on the *R. felis* chromosome, and their distribution in other rickettsiae, other bacterial sequences, and other non-bacterial taxa.

pRF and pRFδ (18)				Distribution b												
ORF a	Name	Annotation	%GC	AG			TG		TRG		SFG			bac	nb	pl
				Br	Bo	Ca	Pr	Ty	Ak	Fe	Ri	Co	Si	y/n/f	y/n	y/n
**pRF01**	*tnp*	hypothetical transposase (or inactive derivative)	0.35	—	—	—	—	—	—	5	—	—	—	y	n	n
pRF02	—	hypothetical transcription regulatory protein	0.26	t	t	r,t	r,t	r,t	r,t	4,r,t	r,t	r,t	r,t	f	n	n
pRF03	*parA*	possible cytokinesis regulatory protein	0.28	1	1	1	1	1	1	3	1	1	1	y	y	n
pRF06	*HsdR*	type I restriction-modification system methyltransferase subunit	0.32	1	1	1	—	t	t2	4,t	—	t2	t2	y	y	n
pRF08	—	similar to a part of CheY-like receiver domain	0.30	1	1	1	1	1	1	3	1	1	1	y	n	n
pRF10	—	rickettsial hypothetical protein	0.30	—	—	1	—	—	1	3	1	1	1	f	n	n
pRF11	*pat2*	patatin-like phospholipase	0.38	r	1	s	2,t	1	1	3	1	1	1	y	n	n
pRF13	*tmk*	thymidylate kinase (TMPK)	0.36	1	1	1	1	1	1	3	1	1	1	y	n	n
pRF41	*tnp*	transposase 31: putative transposase, YhgA-like [pfam04754]	0.34	9,t	12,t	t	—	—	t3	5,t	t3	t	t4	y	n	n
**pRF42**	*ank*	ankyrin-repeat containing gene (ANK)	0.31	—	—	—	—	—	—	3	—	—	—	f	y	n
pRF43	*traDF*	putative conjugative transfer protein TraD (*E. coli* F plasmid)	0.38	1,*	1,*	2,t	*	*	*	2	*	*	*	y	n	n
**pRF51**	*hspP2*	small heat-shock protein 2	0.27	—	—	—	—	—	—	3	—	—	—	y	n	n
pRF52	*hspP1*	small heat-shock protein 1	0.30	t	t	t	t	t	t	3,t	t	t	t	y	n	n
pRF54	*tnp*	transposase, mutator family (transposase_mut) [pfam00872]	0.33	r	1	—	—	—	—	18	—	—	—	y	n	n
**pRF55**	*tnp*	transposase, mutator family (transposase_mut) [pfam00872]	0.33	—	—	—	—	—	—	22	—	—	—	y	n	y
**pRF61**	*tnp*	transposase 31: putative transposase, YhgA-like [pfam04754]	0.36	—	—	—	—	—	—	3	—	—	—	y	n	y
**pRF64**	*tnp*	transposase 14 [pfam01710]	0.29	—	—	—	—	—	—	3	—	—	—	y	n	y
**pRF65**	—	*R. felis* specific protein	0.26	—	—	—	—	—	—	3, t	—	—	—	f	y	f
**pRF (12)**				**Distribution ** b												
**ORF** a	**Name**	**Annotation**	**%GC**	**AG**			**TG**		**TRG**		**SFG**			**bac**	**nb**	**pl**
				**Br**	**Bo**	**Ca**	**Pr**	**Ty**	**Ak**	**Fe**	**Ri**	**Co**	**Si**	**y/n/f**	**y/n**	**y/n**
pRF16	*tpr*	tetratricopeptide repeat domain (TPR)	0.36	1	1	—	—	—	—	6	—	—	—	y	y	n
pRF17	*tpr*	tetratricopeptide repeat domain (TPR)	0.35	2,r	2	—	—	—	—	4	r	r	—	y	y	n
pRF18	*tpr*	tetratricopeptide repeat domain (TPR)	0.36	1	1	—	—	—	—	6	—	—	—	y	n	n
pRF19	—	chromosomal replication initiator protein DnaA-like protein	0.34	1,t	1,t	t2	t	t	t	2,t	t2	t	t	f	y	n
pRF23	*parA*	possible cytokinesis regulatory protein	0.32	—	—	—	—	t	—	2,t	—	—	—	y	y	n
pRF25	*sca12*	cell surface antigen 12	0.41	1	2	1	1	2	5	5	3	2	2	y	n	n
pRF26	*lon*	ATP-dependent protease La, bacterial type (TPR-containing)	0.35	1	1	1	1	1	1	2	1	1	1	y	n	n
pRF27	—	similar to ABC_SMC_euk (chromosome maintenance)	0.36	r	r	—	r	r	—	2,r	—	—	—	f	y	n
pRF29	—	rickettsial hypothetical protein	0.35	—	—	—	—	—	—	2,t	t	—	—	f	y	y
pRF30	*tnp*	transposase, mutator family (transposase_mut) [pfam00872]	0.34	r	r	—	—	—	—	14,t	—	—	—	y	n	n
pRF31	*tnp*	COG3328: transposase (or inactive derivative)	0.31	—	1	—	—	—	1	20	—	—	—	y	n	n
pRF35	*parB*	cleaves ssDNA and supercoiled plasmid DNA	0.36	r	r	r	r	r	r	2,r	r	r	r	y	n	n

a ORF labels that are bolded depict putative genes that are unknown from other published rickettsiae genomes. Underlined ORFs depict sequences analyzed in Figures 2B and 3.

b AG = ancestral group, TG = typhus group, TRG = transitional group, SFG = spotted fever group, bac = present (y) or absent (n) in a diverse array of non-rickettsial bacteria, or present in a few (f) bacteria, nb = present (y) or absent (n) in non-bacterial organisms. Presence (y) or absence (n) of putative orthologs found in other plasmids (pl) are listed. Br = *R. bellii* str. RML369-C, Bo = *R. bellii* str. OSU 85 389, Ca = *R. canadensis* str. McKiel, Pr = *R. prowazekii* str. Madrid E, Ty = *R. typhi* str. Wilmington, Ak = *R. akari* str. Hartford, Fe = *R. felis* str. URRWXCal2, Ri = *R. rickettsii*, Co = *R. conorii* str. Malish 7, and Si = *R. sibirica* str. 246. r = reduced gene relative to the plasmid gene, t = truncated gene relative to the plasmid gene, s = split gene relative to the plasmid gene. * = similar to virD4 genes.

Second, pRF may exist as a resistance, or *(R)plasmid*, that can allow a tolerance for antibiotics or poisons present in the niche of *R. felis*. While there is an elevated level of drug resistance genes on the *R. felis* chromosome [22], including six *R. felis*-specific proteins, there are no genes on pRF that suggest the plasmid plays any role in antibiotic or poison evasion. Third, pRF could function as a *Col-plasmid*, killing other bacteria with colicine gene products (bacteriocins). So far, no colicine gene orthologs have been identified in any rickettsiae genomes. Fourth, because 17 of the genes on the pRF plasmid have not been characterized (or annotated), it cannot be ruled out that pRF isn't a *degrative plasmid*, enabling the digestion of unusual substances like toluene or salicylic acid, which may be encountered in primary and secondary host environments.

Finally, pRF could be a *virulence plasmid*, allowing the bacterium to be pathogenic to its primary and secondary hosts. As a prerequisite to virulence, pathogenic bacteria must have proteins involved in recognition of and adaptation to host cells. pRF contains both ANK (pRF14, pRF42) and TPR (pRF12, pRF15, pRF16, pRF17, pRF18, pRF24, pRF26) motif-containing genes that are typically involved in protein-protein interactions in eukaryotic cells [46], [47], [48], [49], [50], [51], [52]. ANK-containing genes are known from other intracellular pathogens such as *Wolbachia pipientis* [53], *Ehrlichia phagocytophila* [47], *C. burnetii* [54] and *Legionella pneumophila* [51] and likely play an important role in the manipulation of host cell physiology. In addition, one copy of a surface antigen, *sca12* (pRF25), is also present on pRF. Previously, two pRF genes were implicated as likely virulence factors [22]: a patatin homolog (pRF11), *pat2*, which is a patatin-like phospholipase, and a hyaluronidase gene (pRF56), which codes for an enzyme, or “spreading factor”, that increases host tissue permeability and enables antigenic disguise. BLASTP results suggest that *pat2* is found in other α-proteobacteria as well as plants (Table S1), while the putative hyaluronidase is similar to distantly related bacteria as well as proteins from various arthropod genomes (Table S2). These proteins are strong candidates for testing the role of pRF in virulence. Furthermore, they may provide the best explanation for why *R. felis* is the only known *Rickettsia* species that has retained plasmids. Pat2 of pRF has been shown to be unrelated to other patatins present on the *R. felis* chromosome and the chromosomes of other rickettsiae [22], [55], and, although these patatins have been ruled out as factors for phagosomal escape [56], an ascribed phospholipase activity and conservation of A_2_ active sites suggests that Pat2 likely has a role in *R. felis* virulence that is distinct from other rickettsiae. Interestingly, the pFra plasmid of *Yersinia pestis*, the causative agent of plague, encodes a phospholipase D gene, *Ymt*, that is essential for colonization of the flea midgut and eventual transmission to a secondary mammalian host [57], [58]. This virulence plasmid is absent in the species *Yersinia pseudotuberculosis*, which is the less virulent immediate ancestor to *Y. pestis* [59], [60]. Additionally, the presence of a hyaluronidase gene on pRF, which is not found in other rickettsiae, suggests an important role in the life cycle of *R. felis*. These two genes may be critical to the survival and virulence of *R. felis* such that their failure to integrate into the chromosome has driven the retention of plasmids in *R. felis* (and other as yet unidentified plasmid-containing *Rickettsia* spp.). Thus, it is probable that some genes on pRF confer *R. felis* with the ability to recognize, invade and cause virulence in host cells. This is consistent with another bacterial pathogen, *Bacillus anthracis*, the causative agent of anthrax, that seemingly acquired virulence plasmids from a source other than its most common ancestor, which lacks plasmids [61]. Like *B. anthracis* and *Y. pestis, R. felis* could have acquired its pRF plasmid in a serendipitous event that allowed for colonization of a new primary host (flea) as well as a range of secondary vertebrate hosts, a mechanism recently proposed to explain “host jumping” in pathogenic bacteria [62]. This hypothesis is certainly consistent with the unique combination of SFG and TG rickettsiae characteristics that define *R. felis*.

Further support for a functional plasmid that is probably essential for the life cycle of *R. felis* comes from analysis of other interesting proteins on pRF that have plasmid-like characteristics. For instance, there are 12 ORFs on pRF that are similar to transposase (*tnp*) genes from 10 different transposase (or inactive derivative) families (Tables 1 and 2). This high occurrence of transposases suggests that pRF genes have been frequently rearranged through recombination mediated by *tnp* elements. This is further supported by the presence of two putative integrase genes (pRF14, pRF42), which typically integrate viral DNA into host chromosomes, a putative TnpR resolvase gene that codes for a site specific recombinase that is typical of plasmids (pRF32), a putative *lon* gene coding for an ATP-dependent serine protease La (bacterial type) (pRF26) likely involved in DNA-binding and cytokinesis, three copies of a DnaA-like protein (pRF05, pRF19, pRF20) that could function in initiation of plasmid replication, and a site-specific recombinase with similarity to DNA invertase Pin homologs (pRF66). Two *parA* genes (pRF03, pRF23) on the plasmid could be involved in cytokinesis, and one *parB* gene (pRF35) is likely responsible for cleaving ssDNA and particularly super-coiled plasmid DNA. Finally, pRF22 is similar to the plasmid Ppsr1 ORF 12 from *Pseudomonas syringae* [63].

Some plasmid-containing bacteria have incorporated toxin-antitoxin (TAT) systems for keeping the partitioning and inheritance of plasmids stable [64], [65], [66], [67]. Under constitutive expression with the antitoxin component on the plasmid and the toxin on the chromosome, the lethality of the more stable toxin is mediated by the unstable antitoxin. Upon faulty segregation of plasmids after cell division, plasmidless daughter cells are killed by elevated toxin levels due to the breakdown of the unstable antitoxin [68], [69], [70]. Aside from the 16 toxin and 14 antitoxin genes identified in the *R. felis* chromosomal genome, we suggest that another ORF (RF1343) may encode a putative toxin with mild homology to the *mazF* toxin gene of several plasmid-containing bacteria. The *mazEF* TAT module, first characterized in *E. coli* [71], [72], is found on the chromosomes of many bacteria and functions in programmed cell death [73]. Interestingly, a BLAST search using RF1343 as the query resulted in two other *Rickettsia* spp. containing this putative toxin: *R. bellii* str. RML369-C and *R. akari*, suggesting that all other rickettsiae have lost this gene. Alternatively, this putative *mazF* toxin gene could have been transferred from the AG rickettsiae to TRG rickettsiae. No other α-proteobacteria seem to contain a *mazF* toxin gene, and the most similar homologs are from three species of Firmicutes and one β-proteobacterium, *Ralstonia eutropha* str. H16 (Table S3). Searches for a complementary antitoxin *mazE*-like protein in other rickettsiae were unsuccessful. While TAT systems are found in many free-living bacteria, they are rare among obligate intracellular pathogens [74], [75]. The presence of RF1343 in the *R. felis* genome may hint at an evolutionary relic of a once functional system for regulating programmed cell death in the absence of stable plasmid inheritance [76]. Furthermore, the retention of only one component of this TAT module could allude to a switch in function of the *mazF* toxin homolog for adaptation to eukaryotic hosts, as has been suggested for other *R. felis* toxin and antitoxin genes [22]. Such “neofunctionalization” [77] could likely be true for many of the duplicate genes acquired laterally in *R. felis* that have not been subjected to decay in the midst of selection favoring the original ortholog. Interestingly, another component of the *maz* system, *mazG*, whose gene product functions as a nucleoside triphosphate pyrophosphohydrolase, is present and extremely variable across rickettsiae (data not shown).

### Identification of pRF *oriV* and replication termination regions

**Figure 4:**
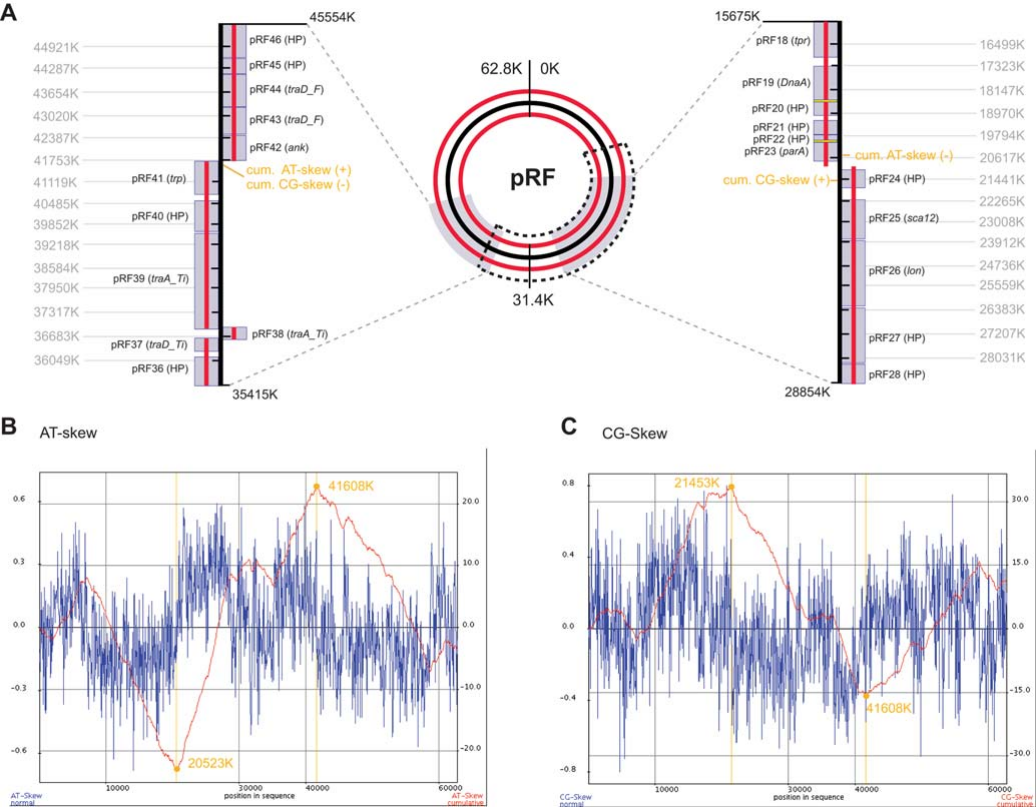
Characteristics and summary information of predicted origin of replication (*oriV*) of the pRF plasmid of *Rickettsia felis.* (A) Schematic map of the pRF with shaded regions containing the putative *oriV* (right) and replication termination region (left). The region outlined in the dark dashed line depicts the portion of the plasmid missing in pRFδ (pRF15-pRF38). Grey boxes depict genes, with gene names described in Tables 1 and 2. Red lines depict coding strands, and yellow blocks depict areas of gene overlap. (B) AT-skew of pRF, with AT-skew (blue), cumulative AT-skew (red) and minimum AT-skew (orange). (C) CG-skew of pRF, with CG-skew (blue), cumulative CG-skew (red) and maximum CG-skew (orange). Plots generated and values computed with GenSkew (http://mips.gsf.de/services/analysis/genskew).

We identified the putative origin of replication (*oriV*) of pRF (Figure 4) based on results from GenSkew (http://mips.gsf.de/services/analysis/genskew), an application for computing and plotting nucleotide skew data. It has previously been reported for *Borrelia burgdorferi* linear and circular plasmids that the *oriV* maps to regions of the plasmids wherein a significant and pronounced switch in DNA strand compositional asymmetry (AT and CG skew) occurs [78]. We have identified the minimum cumulative skew of pRF at positions 20523K (AT-skew) and 21453 (CG-skew) which occurs within pRF23 (*parA* homolog) and pRF24 (hypothetical protein) (Figure 4). ParA functions in cytokinesis and replication initiation [79], and a BLASTP search using pRF23 retrieved with high homology other identified ParA proteins and orthologs with predicted functions in DNA replication and plasmid partitioning (Table 3). Additionally, within a ten-gene range from the pRF23 gene we found two other genes coding for putative Lon (pRF23) and DnaA (pRF23) proteins whose functions in replication are described above. The putative region of replication termination is marked by the minimum CG- and maximum AT-skews (Figure 4) and, rather interestingly, is flanked on both the 5′- and 3′- sides by five of the seven genes associated with the conjugation apparatus (the other two genes are close on the 3′-end). Thus it is highly likely that we have determined the *oriV* and replication termination regions, providing more evidence that pRF is indeed a functionally-replicating plasmid.

**Table 3:** Results of a BlastP search using pRF23 (parA) as a query.

Accession no.	Taxon/annotation	score (bits)	E value
NP_940697.1	*Pseudomonas syringae* pv. *syringae*; stability protein	229	5e-59
NP_114201.1	*Pseudomonas syringae* pv. *maculicola*; ParA	228	2e-58
YP_245407.1	*Actinobacillus porcitonsillarum*; ParA-like	221	2e-56
YP_245399.1	*Actinobacillus porcitonsillarum*; ParA-like protein	218	2e-55
NP_053131.1	*Escherichia coli*; HP pB171_069201	201	1e-50
YP_190184.1	*Escherichia coli*; putative plasmid partitioning protein	201	2e-50
YP_454299.1	*Sodalis glossinidius* str. ‘morsitans’; HP SG0619	163	4e-39
BAD83724.1	*Moraxella bovis* Epp63; putative partition protein	146	6e-34
ZP_00518773.1	*Crocosphaera watsonii* WH 8501; Cobyrinic acid a,c-diamide synthase	144	2e-33
YP_379033.1	*Chlorobium chlorochromatii* CaD3; HP Cag_0719	133	4e-30
ZP_00414461.1	*Arthrobacter* sp. FB24; Cobyrinic acid a,c-diamide synthase	131	2e-29
YP_308764.1	*Escherichia coli* HP LH0102	103	5e-21
ZP_00838506.1	*Shewanella* sp. PV-4; conserved HP	94.0	3e-18
ZP_00814877.1	*Shewanella putrefaciens* CN-32; conserved HP	92.4	1e-17
ZP_00851578.1	*Shewanella* sp. ANA-3; conserved HP	91.7	2e-17
NP_718386.1	*Shewanella oneidensis* MR-1; HP SO2808	91.3	2e-17
YP_734556.1	*Shewanella* sp. MR-4; hypothetical protein Shewmr4_2428	90.5	4e-17
NP_936926.1	*Vibrio vulnificus* YJ016; HP VVA0870	90.1	5e-17
ZP_00582484.1	*Shewanella baltica* OS155; conserved HP	89.4	1e-16
YP_562459.1	*Shewanella denitrificans* OS217; HP Sden_1451	87.8	3e-16
ZP_00586925.1	*Shewanella amazonensis* SB2B; conserved HP	85.5	1e-15
YP_751114.1	*Shewanella frigidimarina* NCIMB 400; HP Sfri_2431	85.1	2e-15
YP_516224.1	*Sodalis* phage phiSG1; HP SGPHI_0046	83.2	6e-15
YP_665408.1	*Helicobacter acinonychis* str. Sheeba; ParA	80.1	6e-14
ZP_01132364.1	*Pseudoalteromonas tunicata* D2; parA family protein	80.1	6e-14

Only sequences with a score greater than 80 bits are shown.

### Second *R. felis* Plasmid, pRFδ?

Ogata et al. [21], [22] identified a second plasmid of 39,263 bp in *R. felis*, pRFδ, which is identical to pRF except for the deletion of 24 contiguous genes, pRF15 to pRF38 (Figure 4A). Of these deleted genes, 12 are exclusive to pRF (Table 1), while the other 12 are present on both the *R. felis* chromosome and pRF (Table 2). We call into question the existence of pRFδ for five reasons. First, several of the important genes predicted to be involved in plasmid maintenance and replication are absent in pRFδ, including genes coding for two putative Dna-like chromosomal replication initiator proteins (pRF19 and pRF20), the putative cytokinesis regulatory protein ParA (pRF23), a putative structural maintenance of chromosomes protein (ABC_SMC_euk) (pRF27), and ParB, a protein implicated in the cleavage of ssDNA and supercoiled plasmid DNA (pRF35). Second, six pRF proteins that have homology to proteins from other plasmid-containing bacteria are absent in pRFδ, including the ORF similar to *P. syringae* plasmid Ppsr1 ORF12 (pRF22), rickettsial hypothetical protein pRF29, the plasmid-encoded site specific recombinase TnpR (pRF32), a DNA polymerase III epsilon subunit-like protein with WGR domain (pRF34), and the putative conjugative transfer proteins TraD Ti (pRF37) and TraA Ti (pRF38). Third, our recent attempts (unpublished data) and those of Pornwiroon et al. [80] to amplify pRFδ in a different strain of *R. felis* (str. LSU) were unsuccessful. Fourth, our predicted *oriV* of pRF that is substantiated by gene composition, sharp change in coding strand and nucleotide compositional skew, is deleted in pRFδ (Figure 4A), suggesting that another means of plasmid replication would be responsible for its continual inheritance. Finally, the fifth reservation we have with the existence of pRFδ in *R. felis* deals with plasmid incompatibility. Plasmid incompatibility is the failure of two co-resident plasmids to be stably inherited without external selection [81]. Incompatibility arises either by conflict in common replication or maintenance elements found in each unique plasmid, or by interference with the ability to correct stochastic fluctuations in copy number of the co-resident plasmids [82]. Even though pRFδ is lacking several of the important genes suspected in plasmid replication and maintenance, the presence of other genes identical to pRF would likely result in plasmid incompatibility, either by conflict in maintenance, replication or regulation of copy number. Collectively, these five points suggest that pRFδ may be an artifact of the genome sequencing and assembly procedures, and that only one plasmid, pRF, probably occurs in strains of *R. felis*.

### Horizontal Transfer of pRF Genes

Likely due to their highly reductive genomes [83], [84], [85], there is relatively little HGT within α-proteobacteria [86], and particularly rickettsiae [43], [87], [88]. This is consistent with other parasitic bacteria with small genomes, such as *Mycoplasma genitalium* and *Chlamydia* spp. [89], [90] and the actinobacterium *Tropheryma whipplei* [91]. Some examples of HGT between α-proteobacteria and their hosts are well known. For instance, segments of the genome of a *Wolbachia* endosymbiont (*w*Ovo) have been inserted into the nuclear genome of its host, a human-parasitic nematode, *Onchocerca volvulus* [92]. Similarly, a large segment of DNA from a *Wolbachia* endosymbiont of the aduki bean beetle, *Callosobruchus chinensis*, has been detected on the X chromosome in the nuclear genome of the beetle [93]. Still, plasmids are thus far unknown from other Rickettsiales, suggesting other means for genetic exchange between *Wolbachia* spp. and their hosts. HGT between chlamydiae and rickettsiae has been suggested for the transfer of ADP/ATP-translocase genes (*tlc*) from the former to the latter roughly 1.5 billion years ago while both organisms likely inhabited single-celled primitive eukaryotes [94], [95]. Regarding the hypothetical immediate ancestor to TG and SFG rickettsiae, HGT has been proposed for the acquirement of S-adenosylmethionine synthetase (*metK*) from an unknown γ-proteobacterium [96]. Recently, the genome sequence of *R. bellii* str. RML369-C revealed the presence of many genes with high homology to several obligate intracellular symbionts of amoebae, suggesting that at one time *R. bellii* likely exchanged genetic information, particularly with the ancestors of *Legionella* and *Protochlamydia*, while residing in an amoeba-like ancestral protozoan [15]. While the *R. bellii* str. RML369-C genome encodes a complete set of putative conjugal DNA transfer genes (Tables 1 & 2), the lack of plasmids suggests that either *R. bellii* once had plasmids and has lost them or integrated them into its genome, or that *R. bellii* possesses the ability to naturally uptake DNA from its environment. Regardless, it is certainly plausible to suggest that *R. bellii* and *R. felis* have had the capability to exchange DNA either by conjugation or some other as of yet uncharacterized mechanism.

While the extent to which the putative conjugation proteins of pRF function in the lateral exchange of genes is still a mystery, it is probable that some degree of HGT has occurred between pRF and other bacteria, particularly AG rickettsiae and other distantly related bacteria. It is well known that plasmids function as vehicles for DNA transfer, and the characterization of the composition of pRF described above implicates as many as 32 of the total 68 pRF genes as putative candidates for HGT (Tables 1 & 2). These 32 genes are not found in other rickettsiae, yet 18 of them are present with high homology in other bacterial genomes, and 14 share limited similarity with non-bacterial sequences. Interestingly, 13 of the total 68 pRF genes are similar to proteins present on plasmids in other bacteria, suggesting that their origin may be from other distantly-related plasmid-containing bacteria. We calculated the %GC for each pRF gene to determine if a significant deviation from the average base composition of the plasmid or *R. felis* chromosome exists (Table 1 & 2). Within a range (±5) of the base composition of the *R. felis* genome (32.5% GC), only seven pRF genes deviate from the average base composition (pRF02, pRF04, pRF14, pRF25, pRF51, pRF65, pRF67). None of these genes are known from plasmids of other bacteria, and it is likely that they are just extremes to the average base composition of the *R. felis* genome. However, absence of base compositional bias alone should not be used to rule out HGT, as the base composition of transferred DNA segments will likely become nearly identical to the host genome over time [97], [98], [99], especially in intracellular symbionts wherein elevated mutation rates are typical [100].

Many bacterial genes passed horizontally likely fail at becoming an integral component of the new genome, with their eventual loss of function and subsequent decay into pseudogenes [43], [101]. Only two pRF proteins are unique to *R. felis* (pRF09 and pRF59), having no putative function or orthology with other known proteins. This could, again, be a consequence of the highly reductive genomes of rickettsiae, or it could reflect two genes that have recently been laterally acquired and have yet to undergo decay. BLAST searches against the public virus protein database recovered no similarity between these ORFs and viruses, supporting the recent proposal that proteins with no recognizable homology to any other life are less likely to have been acquired from viruses than are other characterized proteins [102].

### Chromosomal Virulence Factors Derived from Primitive Plasmids

The number of intracellular pathogens that have acquired the conjugal transfer systems of plasmids and modified them for the export of toxins is growing [103], [104]. The presence of putative type IV pili in *R. felis* could imply that this machinery is responsible for the secretion of virulence factors and other proteins involved in host tropism, as was recently determined for *Francisella tularensis* virulence [105]. Alternatively, a modification of a second “adapted” T4SS could be involved in the shunt of effectors into eukaryotic host cells. This is at least the more likely scenario as other plasmidless rickettsiae seem to have the ability to secrete effector molecules directly into host cells [106]. The machinery for bacterial T4SS has been suggested to have undergone a great amount of HGT [77]. Phylogenetic analysis implies that the ancestors to all chromosomally encoded T4SSs are plasmid-derived, with the lateral transfer of plasmid-encoded genes to the chromosome much more likely than vice versa [77]. This supports the hypothesis that all rickettsiae may have once harbored plasmids, with T4SS components derived from these plasmids, either through independent acquirement events or one event ancestral to all rickettsiae. Indeed, the putative *TraDF* gene (pRF43) shares close similarity with the *virD4* genes of other rickettsiae (Table 2). Interestingly, AG rickettsiae contain additional copies of *virD4* that are more similar to pRF43 than to the other non-AG rickettsiae *virD4* genes, further strengthening the argument for HGT between *R. felis* and AG rickettsiae.

In theory, the presence of duplicated T4SS genes would allow for bacteria to continue the use of pili-mediated conjugation with a separate adapted conjugation system for the secretion of virulence factors. The only two *Rickettsia* spp. known to have conjugative pili are *R. felis* str. URRWXCal2 and *R. bellii* str. RML369-C [15], [21], [22]. Not surprising is that these two genomes also contain elevated copies of transposable elements, insertion sequences and repetitive elements relative to other sequenced rickettsial genomes [107]. They also contain more HGT candidates than other rickettsiae, particulalry genes likely inherited from Legionellaceae and Parachlamydiaceae [15]. While *R. felis* seemingly has the ability to transfer plasmids via its pilus, the genome of *R. bellii* str. RML369-C does not harbor plasmids by which to exchange DNA. However, conjugative transposons are also efficient at transferring DNA through the pilus [108]. Indeed, another intracellular pathogen, *Legionella pneumophila*, performs T4SS-mediated conjugative transfer of both chromosomal [109] *and* plasmid [110] DNA. Thus, even in the absence of plasmids, rickettsiae with pili are likely able to exchange DNA via conjugation with other bacteria, as well as uptake genetic material from non-bacterial organisms.

In rickettsiae, no proteins potentially involved in virulence have been demonstrated to be secreted via the T4SS. However, we predict that some rickettsiae T4SS effectors characterized in the future will be genes inherited horizontally from distantly-related organisms, as products of HGT in prokaryotic genomes are often associated with pathogenicity [111]. For example, HGT has been implicated in the acquirement of bacterial α_2_-macroglobulins, which function as colonization factors, in many diverse bacteria exploiting higher eukayotes as hosts [112]. The recently sequenced genome of *Chlamydophila abortus* revealed several highly variable genomic islands, some of which contain genes involved in host invasion and virulence [113]. Waterfield et al. [61] have demonstrated that a high level of similarity exists between pathogenicity islands from insect and mammalian pathogens. While no pathogenicity islands have been described from rickettsiae, it is likely that virulence factors could have resulted from previously acquired pathogenicity islands that are difficult to detect in highly reductive genomes. For instance, the presence of gene clusters involved in virulence or immunity on plasmids are often flanked by repetitive and direct repeats [114], as is known for microcin C51 plasmid genes of *E. coli* that are believed to be laterally inherited [115]. Some of these genes are present in various rickettsiae in truncated and highly variable copies (data not shown). One microcin, *mccE*, is similar to a rickettsial gene that codes for *rimJ*, a ribosomal-protein-alanine N-acetyltransferase. While conserved across all rickettsiae, *rimJ* is unknown from other Rickettsiales, with the closest putative orthologs found in various genomes of *Coxiella* and *Bacillus* (Table S4). Even if the similarity between *rimJ* and *mccE* is the result of convergence in the C-terminal catalytic domain, the close affinity of *rimJ* to other bacteria well diverged from rickettsiae, many of which harbor plasmids (Table S4), may hint at the horizontal inheritance of this gene in rickettsiae. Also, one of the above-mentioned pRF genes possibly involved in *R. felis* virulence, the hyaluronidase (pRF56), has close similarity with several proteins from insects, suggesting it could be derived from an insect host gene and is involved in host manipulation (Table S2).

The search for virulence factors involved in rickettsial pathogenicity has not been easy and will entail intense scrutiny of the predicted ORFs with unknown function across the growing number of rickettsial genomes. Due to their streamlined evolution with constraints on genome size relative to many other bacteria, the detection of HGT in rickettsiae will be difficult for the following reasons: 1) most of the introduced genes are likely rapidly degraded and pruned from the genome, 2) accelerated rates of nucleotide evolution quickly disguise horizontally-inherited genes by homogenizing overall genomic base composition, and 3) neofunctionalization of co-opted genes only retains those motifs important in structure and function, making homology assignment virtually undetectable for similarity algorithms. Nevertheless, through an understanding of these properties of rickettsial evolution, coupled with rigorous phylogeny estimation and *in vitro* characterization, will a repertoire of horizontally inherited virulence factors begin to emerge, further illustrating the means by which these parasitic bacteria manipulate hosts cells throughout their life stages.

### Plasmid Plasticity and Associated Virulence Traits

Of the 53 sequenced α-proteobacterial genomes currently available on the NCBI microbial genome database (http://www.ncbi.nlm.nih.gov/genomes/lproks.cgi), 16 are known to contain plasmids. Aside from *Rickettsia*, two other genera have species both with and without plasmids, *Nitrobacter* and *Rhodopseudomonas*, suggesting that plasmids are not essential for all species within α-proteobacterial genera that contain them. This plasticity for harboring plasmids is further intricate when analyzing intraspecific plasmid number. Three α-proteobacterial species differ in the number of plasmid types within their genomes: *Mesorhizobium* (2–3), *Rhizobium* (5–6) and *Silicibacter* (1–2). Thus it appears typical for at least α-proteobacterial plasmid-containing species that plasmids can be present or absent, and that the number of plasmid types per genome is plastic. This pattern extends to include four other genera of obligate intracellular pathogens (Table 4). It seems that plasmids are plastic across all five genera of the obligate intracellular pathogens listed, and in the instances where virulence factors are associated with the plasmid, plasmidless strains have incorporated the essential genes into their chromosomes (35,37,38,39,40). Thus, even though laboratory methods for detecting plasmids, such as pulse-field gel electrophoresis or PCR, may rule out the presence of plasmids within genomes, plasmids may reside within chromosomes either permanently or temporarily, not unlike the behavior of the bacteriophage lambda. Still, integration of plasmids into chromosomes is considered rare, and transfer of plasmid genes to the chromosome is likely the most efficient means for avoiding deleterious mutations or elimination by unequal segregation of important plasmid genes [89].

**Table 4:** Comparative genomic analysis of five plasmid-containing genera of obligate intracellular pathogens.

Taxon	Genome size (MB)	% GC	Plasmid(s)	Plasmid size (MB)	% GC	ORFs	RNAs
*Chlamydia*							
*C. muridarum* Nigg	1.08	40.3	1, pMoPn	0.007501	35.7	7	0
*C. trachomatis* A/HAR-13	1.05	41.3%	1, Pcta	0.00751	36.3	8	0
*C. trachomatis* D/UW-3/CX	1.04	41.3	0	—	—	—	—
*Chlamydophila*							
*C. caviae* GPIC	1.18	39.2%	1, pCpGP1	0.007966	33.7	7	0
*C. felis* Fe/C-56	1.17	39.4%	1, pCfe1	0.00755233.9	8	0	
*C. pneumoniae* AR39	1.23	40.6%	0^1^	—	—	—	—
*C. pneumoniae* CWL029	1.23	40.6%	0^1^	—	—	—	—
*C. pneumoniae* J138	1.23	40.6%	0^1^	—	—	—	—
*C. pneumoniae* TW-183	1.23	40.6%	0^1^	—	—	—	—
*C. psittaci*	NA	NA	1^1^, pAP'p	NA	NA	NA	NA
*Coxiella*							
*C. burnetti* RSA 493	2.03	42.7%	1^2^, pQpH1	0.037393	39.3	36	0
*Mycobacterium*							
*M. leprae* TN	3.27	57.8	0	—	—	—	—
*M*. sp. MCS	5.92	68.5	1, plasmid1	0.215075	66.6	224	0
*Rickettsia*							
*R. bellii* RML369-C	1.52	31.7	0	—	—	—	—
*R. bellii* OSU 85 389	1.52	31.0	1^3^, ?	0.048775(?)	?	49(?)	?
*R. canadensis* McKiel	1.16	29.0	0	—	—	—	—
*R. prowazekii* Madrid E	1.11	28.9	0	—	—	—	—
*R. typhi* Wilmington	1.11	29.0	0	—	—	—	—
*R. akari* Hartford	1.23	32.3	?	?	?	?	?
*R. felis* URRWXCal2	1.59	32.5	1, pRF	0.062829	33.6	68	0
*R. rickettsii*	1.26	32.4	0	—	—	—	—
*R. conorii* Malish 7	1.27	32.5	0	—	—	—	—
*R. sibirica* 246	1.25	32.4	0	—	—	—	—

1 Majority of *C. psittaci* and some *C. pneumoniae* strains carry a plasmid [116], [117], [118].

2 Plasmidless strains of *C. brunetti* occur, with the plasmid incorporated into the chromosome [37], [38].

3 As recently reported [128] (see Conclusion).

Plasmids are also plastic when analyzed across the genome sequences of five other selected pathogenic bacteria, namely *Bacillus, Legionella, Neisseria, Pseudomonas* and *Yersinia* (Table S5). Except for *Yersinia*, all five genera contain both strains with and without plasmids, and aside from *Neisseria* and *Legionella*, the number of plasmid types per species is variable. Although three sequenced genomes of *Neisseria* revealed no presence of plasmids, a larger sampling of several pathogenic and commensal neisseriae uncovered six plasmid types, with some containing genes with moderate homology to the recently identified meningococcal disease-associated phage [99]. The virulence traits associated with the plasmids of *Bacillus* and *Yersinia* were discussed above and raise questions as to whether or not plasmidless strains have the genes necessary for virulence incorporated into their chromosomes. Analysis of two close strains of *Legionella pneumophila*, Paris and Lens, revealed three variable plasmids, with only the Paris strain harboring a T4SS encoded on a multicopy plasmid [51]. Interestingly, robust sampling of *L. pneumophila* populations exposed that some genomes have this T4SS-plasmid integrated into the chromosome [51]. Similarly, of three strains of *Pseudomonas*, pv. *tomato* DC3000, pv. *phaseolicola* 1448A and pv. *syringae* B728a, only the first two contain plasmids, with two plasmid types per genome [119], [120], [121]. Analysis of the plasmidless str. pv. *syringae* B728a with other pseudomonad genomes revealed a genomic island with high similarity to a conjugative plasmid from *P. aeruginosa* str. PAO1, pKLC102, which carries a *pil* operon encoding type IV sex pili, as well as recombination and replication machinery [120], [122]. Regarding *Pseudomonas* pv. *tomato* DC3000, even though copies of virulence genes are present on pDC3000A, the plasmid is not involved in virulence, as copies of the virulence genes have functional paralogs on the chromosome [119]. Thus plasmids may initially be involved in virulence, but once the virulence genes are inserted into the chromosome, the plasmid genes are no longer essential for fitness. However, mutational analysis of T4SS genes on the plasmid pVir of *Campylobacter jejuni* str. 81–176 identified five genes that affect *in vitro* invasion of intestinal epithelial cells, suggesting the plasmid is essential for host colonization [123]. Similarly, O'Connell and Nicks [124] demonstrated that plasmid-cured strains of *Chlamydia muridarum* grew smaller plaques than the wildtype and were unable to accumulate glycogen within intercytoplasmic inclusions. Thus, while plasmid plasticity may hint at a reduced role for plasmids in virulence, particularly when virulence genes are copied on the chromosome, it is likely that many plasmid systems are still essential for survival and/or virulence.

The number of gram-negative pathogenic bacteria that use adapted T4SSs for the export of virulence factors to hosts is growing, e.g. *Agrobacterium tumefaciens, Bartonella tribocorum, Brucella* spp., *Helicobacter pylori, Bordetella pertussis, L. pneumophila, R. prowazekii*, and *Yersinia enterocolitica* [106], [125], [126]. Given that plasmid conjugation machineries are likely the ancestors to many of these modified T4SSs, the role of plasmids in the acquirement of virulence is an exciting area for research, particularly for systems wherein plasmids are plastic, such as rickettsiae. Future analysis of plasmid plasticity and characterization of plasmid-associated virulence traits will hopefully answer an important question: *why do the sequenced genomes of other pathogenic rickettsiae not harbor plasmids?* And more interestingly, *why would R. felis need a plasmid with virulence traits when it has the same core set of genes (e.g., pld, tlyc, rompB, pat, etc.) shared by the other nine sequenced rickettsial genomes, some of which have already been implicated in host adherence and pathogenicity?* Studies demonstrating the differential expression of pRF genes during the life cycle of *R. felis* may lead to the identification of genes involved in host invasion and virulence. Indeed, plasmid gene expression has been shown to be highly variable throughout the life cycle of another intracellular pathogen, *C. trachomatis* [127]. Future studies should also determine if *R. felis* is capable of integrating pRF into its chromosome, and how plasmid-curing affects overall fitness.

### Conclusion

**Figure 5:**
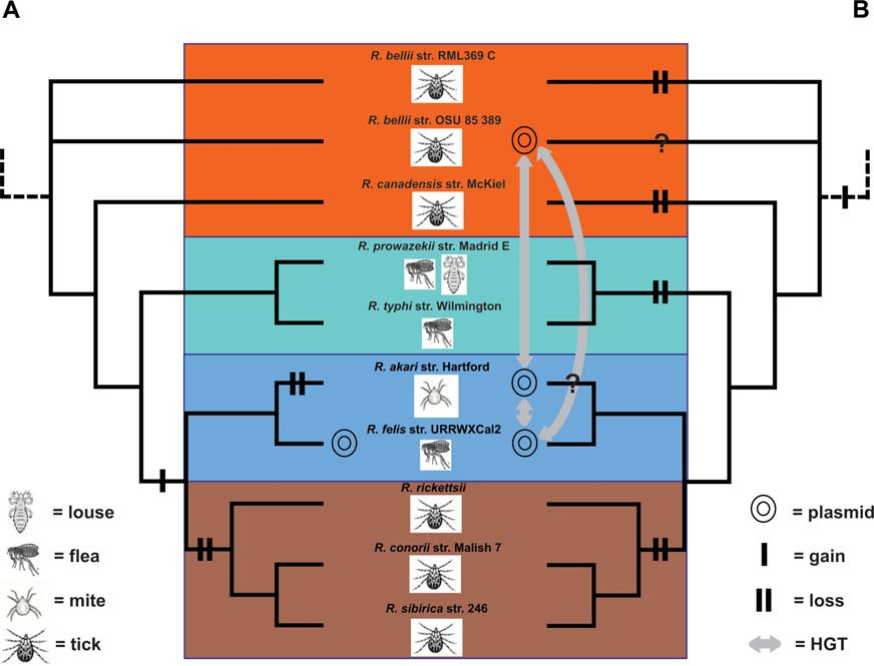
Comparison of two hypotheses for the evolution of plasmids in rickettsiae. (A) The appearance of a plasmid system in *R. felis* (as a member of SFG rickettsiae) as recently suggested (Ogata et al., 2005b; Blanc et al. 2007). (B) Our hypothesis centered on the notion that the ancestor to all rickettsiae harbored a plasmid system with subsequent losses in the ancestors to the TG and SFG rickettsiae, and in *R. canadensis* and *R. bellii* str. RML369-C. Red = ancestral rickettsiae, Aquamarine = typhus group, light blue = transitional group, brown = true spotted fever group. Trees are from Figure 2A.

We have demonstrated above through a phylogenomic/bioinformatic evaluation that there is strong support for the presence of a single plasmid in *R. felis*, pRF, and that many of the plasmid genes have probably been horizontally inherited from exchanges with other organisms either through a rudimentary conjugation apparatus or an adaptation of the T4SS that allows for dual transfer of plasmid DNA as well as translocation of effector molecules. Thus, we doubt the recent proposal that all pRF genes were inherited in one HGT event (Figure 5A), and that this event occurred either in *R. felis* [96] or an ancestor to TRG rickettsiae [21]. Rather, our characterization of the *R. felis* plasmid within a phylogenomic context suggests that the primitive rickettsial ancestor likely harbored plasmids (Figure 5B). In the *Rickettsia* ancestor plasmids would have 1) eased the exchange of DNA with other intracellular pathogens, 2) fostered the duplication of certain genes (especially transposases), creating gene families and new genes through neofunctionalization, 3) facilitated the integration of plasmid genes into the chromosome, and 4) provided for a means to acquire a conjugation operon that would eventually give rise to the T4SS. Under this evolutionary model the plasticity of plasmids can be explained in relation to life history and overall fitness costs associated with plasmids: those lineages with strict host specialization, particularly lineages undergoing extreme gene loss and accelerated rates of nucleotide evolution (i.e., TG rickettsiae), would no longer need to harbor plasmids. Those lineages with a large genome size, elevated numbers of mobile elements and a less strict host range might retain plasmids.

We stress that the discovery of a plasmid system in *R. felis*, as well as the presence of conjugative pili in *R. bellii* and close genomic similarities between the two species, should be considered the opening of Pandora's box, as subsequent completed rickettsial genomes will likely yield more plasmid systems and other means for non-vertical exchange of genetic material within rickettsiae and between rickettsiae and other organisms. For instance, it was recently presented at the annual meetings of The American Society of Rickettsiology [128] that *R. bellii* str. OSU85-389 contains a putative conjugative plasmid (sequence as yet unpublished). Based on this finding, *R. akari* str. Hartford may also contain a conjugative plasmid, as it shares a unique clade (TRG rickettsiae) in the rickettsial tree with *R. felis* that is unique in that it colonizes mite and insect hosts respectively. The presence of a plasmid perhaps allowed for the horizontal acquisition of virulence factors from other mite- and insect-associated endosymbionts that occupy similar niches of TRG rickettsiae. The supposed finding of a plasmid in *R. bellii* str. OSU85-389 is compatible with our analysis of the pRF gene distribution throughout other rickettsiae, as we detected many of the plasmid-specific pRF genes in the genomes of two *R. bellii* strains. Plasmids would undoubtedly be beneficial for *R. bellii* as it has one of the largest host ranges of any *Rickettsia* species.

We predict that as more genomic sequences become available for other *Rickettsia* spp., the four clades defined herein using phylogenetic estimation (AG, TG, TRG, SFG) will remain strongly supported, and that *R. australis* and other rickettsiae with either recent host switches or the presence of plasmids, will likely fall within the AG and TRG rickettsiae. However, because the recently sequenced genome of another member of the SFG rickettsiae, *R. massiliae*, revealed a large genome size and the presence of a *tra* gene cluster similar to that found in *R. bellii* [96], plasmids may be uncovered in other as yet unsequenced SFG rickettsiae. Given the genomic similarities shared between the *R. felis* and *R. bellii* (and likely *R. massiliae*) genomes, it is likely that genetic mobility boosts the versatility and plasticity of these microorganisms. However, the role plasmids play in host colonization and virulence is not well understood, and will likely only become more apparent with both the discovery of plasmids in other rickettsiae, as well as the *in vitro* characterization of the contribution of plasmids to host recognition, invasion and pathogenicity. This aspect of rickettsiology is promising for the future development of vaccines for virulent rickettsiae, as well as for the construction of shuttle vectors for which plasmids may provide the first means for *in vivo* experimental models [23], [129].

## Materials and Methods

### Phylogeny estimation

We analyzed only those *Rickettsia* spp. for which a genome sequence was available: *Rickettsia bellii* str. RML369-C (NC_007940), *R. bellii* str. OSU85 389 (NZ_AARC00000000), *R. canadensis* str. McKiel (NZ_AAFF01000001), *R. prowazekii* str. Madrid E (NC_000963), *R. typhi* str. Wilmington (NC_006142), *R. akari* str. Hartford (NZ_AAFE01000001), *R. felis* str. URRWXCal2 (NC_007109), *R. conorii* str. Malish 7 (NC_003103), *R. rickettsii* (NZ_AADJ01000001), and *R. sibirica* str. 246 (NZ_AABW01000001). Additionally, to root the baseline phylogeny we included orthologous sequences from two outgroup taxa, one from the *Wolbachia* endosymbiont of *Drosophila melanogaster* (NC_002978.6), and one from the *Wolbachia* endosymbiont strain TRS of *Brugia malayi* (NC_006833.1). Methods of phylogeny estimation were the same for generating the baseline phylogeny (Figure 1), the phylogenies of exclusively chromosomal genes (Figure 2A) and those present on chromosomes and the pRF plasmid of *R. felis* (Figure 2B), and the single pRF genes (Figure 3). Initially, BLASTP [130] searches against the NCBI protein database were done using *R. felis* amino acid sequences as queries (for BLASTP specifics and threshold see below). For the baseline phylogeny, we analyzed 15 conserved rickettsial proteins (hypothetical protein RF_0005, threonyl-tRNA synthetase, preprotein translocase SecA subunit, uncharacterized low-complexity protein RF_0864, pyruvate phosphate dikinase precursor, leucyl-tRNA synthetase, hypothetical protein RF_0556, NAD-specific glutamate dehydrogenase, DNA polymerase III alpha chain, O-antigen export system permease protein RfbA, thioredoxin, NADPH-dependent glutamate synthase beta chain and related oxidoreductases, putative TIM-barrel protein in nifR3 family, and UDP-3-O-[3-hydroxymyristoyl] glucosamine). For the exclusively chromosomal dataset, we used the Rickettsia Orthologous Groups database at the PATRIC Website [107] to compile 21 hypothetical proteins that are present in all ten published rickettsial genomes. Our rationale for using hypothetical proteins was that these proteins have never been analyzed in prior studies and thus would test previous phylogeny estimates, as well as evaluate the phylogenetic utility of uncharacterized ORFs. The seven proteins comprising the third dataset consist of genes present of both the *R. felis* chromosome and pRF and distributed across all 10 rickettsial chromosomal genomes. Initially, 10 proteins qualified by this criterion; however, gene duplications, truncations and split genes did not permit the inclusion of pRF02, pRF19 and pRF25 (Table 2). pRF43 was also excluded because of its uncertain homology with chromosomal virD4 genes.

Retrieved rickettsial (and *Wolbachia* outgroup) protein sequences were exported in Fasta format and aligned locally using default parameters in the command-line version of the program MUSCLE [131], [132]. Aligned datasets were converted to Nexus format using the program seqConverter.pl, version 1.1 [133]. Each Nexus file was concatenated manually into a combined executable Nexus file and analyzed under parsimony in an exhaustive search in the program PAUP* version 4.10 (Altivec) [134]. Branch support was assessed using the bootstrap [135] with default settings in PAUP. We performed one million bootstrap replications. Tree images were exported from PAUP* and manually adjusted in Adobe® Illustrator® CS2 v.12.0.1. Single gene phylogeny estimations were performed similarly.

### Characterization and distribution of pRF genes

We performed BLASTP searches against the NCBI protein database using the 68 genes present on the pRF plasmid (NC_007110) as queries. The nr (All GenBank+RefSeq Nucleotides+EMBL+DDBJ+PDB) database was used, coupled with a search against the Conserved Domains Database. Searches were performed across all organisms with composition-based statistics. No filter was used. Default matrix parameters (BLOSUM62) and gap costs (Existence:11 Extension: 1) were implemented, with an inclusion threshold of 0.005. We then compiled the 68 genes into two tables: one based on pRF genes that were present only on pRF (Table 1), and another with genes also found on the *R. felis* chromosome (Table 2). When strong support for a function was given for closely related sequences, we modified the existing annotation for some genes. The presence or absence of the genes across 1) other rickettsiae, 2) other bacteria and 3) other viruses, archaea and eukaryotes was then assigned. For presence/absence in other viruses, archaea and eukaryotes, we did not reject sequences with low homology, as recent studies suggest that regions of divergent proteins sharing limited homology can actually be the result of molecular mimicry [136], [137]. We also determined whether or not pRF genes were present in the plasmids of other bacteria.

### Identification of pRF *oriV*

We predicted the putative origin of replication (*oriV*) of pRF (Figure 4) based on results from GenSkew (http://mips.gsf.de/services/analysis/genskew), an application for computing and plotting nucleotide skew data. We then plotted a ten-gene range from the center of the cumulative AT and GT skews to determine putative *oriV* and replication termination regions through identification of genes known to typically occur in these regions.

All relevant materials, including updates to pRF annotation and sequence alignments used to generate phylogenetic trees, will be available in a future update of the PATRIC rickettsial database [107].

## Supporting Information

Table S1 Results of a BlastP search using pRF11
(0.04 MB DOC)

Table S2 Results of a BlastP search using pRF56
(0.04 MB DOC)

Table S3 Results of a BlastP search using RF1343
(0.04 MB DOC)

Table S4 Results of a BlastP search using RP693
(0.04 MB DOC)

Table S5 Comparative genomic analysis of five pathogenetic bacteria harboring plasmids with associated virulence factors.
(0.07 MB DOC)
