# Supplementary material for: Plasmids and Rickettsial Evolution: Insight from Rickettsia felis
Source: PLoS One. 2007 Mar 7;2(3):e266. doi: 10.1371/journal.pone.0000266 (PMC1800911; doi:10.1371/journal.pone.0000266)
Supplement: Table S1 — Results of a BlastP search using pRF11 (0.04 MB DOC) [file pone.0000266.s001.doc]

**Table S1.** Results of a BlastP search using pRF11 (Pat2) as a query. Only sequences with a score greater than 80 bits are shown.

**Accession no. Taxon/annotation* score E**

**(bits) value**

YP_246376.1 *Rickettsia felis* URRWXCal2; Patatin-like phospholipase 457 6e-127

ZP_00340555.1 *Rickettsia akari* str. Hartford; COG3621: Patatin 438 4e-121

ZP_01347441.1 *Rickettsia canadensis* str. McKiel; HP RcanM_01000354 393 1e-107

ZP_01379747.1 *Rickettsia bellii* OSU 85-389; HP RbelO_01000530 132 4e-29

NP_360559.1 *Rickettsia conorii* str. Malish 7; patatin b1 precursor 129 3e-28

ZP_00153908.1 *Rickettsia rickettsii*; COG3621: Patatin 125 8e-27

ZP_00142926.1 *Rickettsia sibirica* 246; patatin b1 precursor 122 5e-26

NP_220970.1 *Rickettsia prowazekii* str. Madrid E; PATATIN B1 PRECURSOR (pat1) 122 6e-26

YP_067537.1 *Rickettsia typhi* str. Wilmington; patatin-like protein 117 1e-24

NP_966337.1 Wolbachia endosymbiont of Drosophila melanogaster; patatin family protein 114 1e-23

YP_538013.1 *Rickettsia bellii* RML369-C; Patatin-like phospholipase 109 3e-22

ZP_00374057.1 *Wolbachia endosymbiont* of *Drosophila ananassae*; patatin family protein 108 8e-22

ZP_01314725.1 *Wolbachia endosymbiont* of *Drosophila willistoni*; HP Wendoof_01000457 105 7e-21

YP_302831.1 *Ehrlichia canis* str. Jake; Patatin 105 8e-21

NP_568015.1 *Arabidopsis thaliana*; nutrient reservoir 102 7e-20

AAM64566.1 *Arabidopsis thaliana*; patatin-like protein 101 8e-20

NP_001062044.1 *Oryza sativa* (*japonica* cultivar-grp); Os08g0477500 101 8e-20

ZP_01299370.1 *Coxiella burnetii* Dugway 7E9-12; HP CburD_01000492 101 1e-19

CAA05628.1 *Arabidopsis thaliana*; patatin-like protein 100 2e-19

NP_849511.1 *Arabidopsis thaliana*; nutrient reservoir 100 2e-19

NP_195423.1 *Arabidopsis thaliana*; nutrient reservoir 100 2e-19

AAX99411.1 *Gossypium hirsutum*; patatin-like protein 99.8 4e-19

ZP_01118599.1 *Polaribacter irgensii* 23-P; patatin family protein 99.4 4e-19

YP_378344.1 *Chlorobium chlorochromatii* CaD3; patatin family protein 99.0 6e-19

ZP_00544882.1 *Ehrlichia chaffeensis* str. Sapulpa; Patatin 98.6 8e-19

ZP_01254029.1 *Psychroflexus torquis* ATCC 700755; patatin family protein 98.2 1e-18

YP_264656.1 *Psychrobacter arcticus* 273-4; probable patatin-related protein 96.7 3e-18

NP_849512.2 *Arabidopsis thaliana*; nutrient reservoir 95.9 5e-18

YP_569425.1 *Rhodopseudomonas palustris* BisB5; Patatin 95.1 8e-18

YP_673116.1 *Mesorhizobium* sp. BNC1; Patatin 95.1 9e-18

NP_221068.1 *Rickettsia prowazekii* str. Madrid E; HP RP709 94.7 9e-18

AAP70299.1 *Escherichia coli*; VC0178-like protein 94.7 1e-17

YP_322138.1 *Anabaena variabilis* ATCC 29413; Patatin 94.7 1e-17

NP_001062043.1 *Oryza sativa* (*japonica* cultivar-grp); Os08g0477100 94.4 2e-17

ZP_01417352.1 *Sinorhizobium medicae* WSM419; Patatin-like 93.6 3e-17

NP_001062042.1 *Oryza sativa* (*japonica* cultivar-grp); Os08g0476900 91.3 1e-16

AF061282_24 *Sorghum bicolor;* patatin-like protein 90.5 2e-16

AAB08428.1 *Nicotiana tabacum*; patatin homolog 89.7 3e-16

CAC01602.1 *Anabaena circinalis* 90; putative patatin-like protein 88.2 9e-16

YP_721528.1 *Trichodesmium erythraeum* IMS101; Patatin 88.2 9e-16

NP_180224.1 *Arabidopsis thaliana*; nutrient reservoir 87.4 2e-15

AAM63157.1 *Arabidopsis thaliana*; similar to latex allergen from Hevea brasiliensis 86.7 3e-15

ZP_00110907.1 *Nostoc punctiforme* PCC 73102; COG3621: Patatin 86.3 4e-15

NP_001061678.1 *Oryza sativa* (japonica cultivar-grp); Os08g0376500 86.3 4e-15

YP_293212.1 *Ralstonia eutropha* JMP134; Patatin 84.3 1e-14

AF061282_23 *Sorghum bicolor*; patatin-like protein 84.3 2e-14

NP_199172.1 *Arabidopsis thaliana*; nutrient reservoir 84.3 2e-14

AF158253_1 *Nicotiana tabacum*; patatin-like protein 3 83.2 3e-14

NP_001045094.1 *Oryza sativa* (*japonica* cultivar-grp); Os01g0898500 82.8 4e-14

AF061282_2 *Sorghum bicolor*; patatin-like protein 82.0 6e-14

NP_229835.1 *Vibrio cholerae* O1 biovar eltor str. N16961; patatin-related protein 82.0 6e-14

ABA99477.1 *Oryza sativa* (*japonica* cultivar-grp); Patatin-like phospholipase family protein 80.9 2e-13

YP_678618.1 *Cytophaga hutchinsonii* ATCC 33406; patatin-like protein 80.5 2e-13

* HP = hypothetical protein.
