# Supplementary material for: Plasmids and Rickettsial Evolution: Insight from Rickettsia felis
Source: PLoS One. 2007 Mar 7;2(3):e266. doi: 10.1371/journal.pone.0000266 (PMC1800911; doi:10.1371/journal.pone.0000266)
Supplement: Table S2 — Results of a BlastP search using pRF56 (0.04 MB DOC) [file pone.0000266.s002.doc]

**Table S2.** Results of a BlastP search using pRF56 (putative hyaluronidase) as a query. Only sequences with a score greater than 80 bits are shown.

**Accession no. Taxon/annotation* score E**

**(bits) value**

YP_856012 *Aeromonas hydrophila* subsp. hydrophila ATCC 7966; hyaluronidase, putative 201 4e-50

ZP_00817532 *Marinobacter aquaeolei* VT8; conserved HP 199 1e-49

ZP_01102882 gamma proteobacterium KT 71; hyaluronidase family protein 102 3e-20

ZP_01129099 marine actinobacterium PHSC20C1; HP A20C1_09449 100 9e-20

ZP_00604207 *Enterococcus faecium* DO ; Hyaluronidase eukaryotic/prokaryotic 95.5 4e-18

YP_695324 *Clostridium perfringens* ATCC 13124; putative hyaluronoglucosaminidase 93.2 2e-17

NP_561797 *Clostridium perfringens* str. 13; hyaluronidase 92.0 4e-17

XP_966927 *Tribolium castaneum*; PREDICTED: similar to CG5871-PA 91.3 6e-17

ZP_00996755 *Janibacter* sp. HTCC2649; HP JNB_17763 91.3 7e-17

XP_395374 *Apis mellifera*; PREDICTED: similar to CG5871-PA 91.3 8e-17

YP_695887 *Clostridium perfringens* ATCC 13124; putative hyaluronidase 90.9 8e-17

NP_562150 *Clostridium perfringens* str. 13; hyaluronidase 90.5 1e-16

2CBIA *Clostridium Perfringens* ; Nagj Family 84 Glycoside Hydrolase 89.4 2e-16

EAA03784 *Anopheles gambiae* str. PEST; ENSANGP00000006225 87.8 7e-16

XP_307891 *Anopheles gambiae* str. PEST; ENSANGP00000006225 87.4 9e-16

EAT38838 *Aedes aegypti*; conserved HP 83.6 2e-14

YP_198854 *Xanthomonas oryzae* pv. oryzae KACC10331; HP XOO0215 82.4 3e-14

YP_449218 *Xanthomonas oryzae* pv. oryzae MAFF 311018; HP XOO_0189 82.4 3e-14

EAL27188 *Drosophila pseudoobscura*; GA19193-PA 81.3 8e-14

AG10789 *Tetraodon nigroviridis*; unnamed protein product 80.9 9e-14

P_644229 *Xanthomonas axonopodis* pv. citri str. 306; HP XAC3928 80.9 9e-14

P_365774 *Xanthomonas campestris* pv. vesicatoria str. 85-10; putative secreted protein 80.1 1e-13

* HP = hypothetical protein.
