# Supplementary material for: Plasmids and Rickettsial Evolution: Insight from Rickettsia felis
Source: PLoS One. 2007 Mar 7;2(3):e266. doi: 10.1371/journal.pone.0000266 (PMC1800911; doi:10.1371/journal.pone.0000266)
Supplement: Table S3 — Results of a BlastP search using RF1343 (0.04 MB DOC) [file pone.0000266.s003.doc]

**Table S3.** Results of a BlastP search using RF1343 (MazF) as a query. Only sequences with a score greater than 40 bits are shown.

**Accession no. Taxon/annotation* score E**

**(bits) value**

YP_537927 *Rickettsia bellii* RML369-C; Growth inhibitor 128 1e-28

ZP_00340884 *Rickettsia akari* str. Hartford; COG2337: Growth inhibitor 58.2 1e-07

ZP_00605137 *Enterococcus faecium* DO; PemK-like protein 54.3 2e-06

YP_841373 *Ralstonia eutropha* H16; PemK-like protein DNA binding protein 53.9 2e-06

YP_520249 *Desulfitobacterium hafniense* Y51; HP DSY4016 52.4 6e-06

YP_807723 *Lactobacillus casei* ATCC 334; TAT addiction module toxin comp. MazF (an endoRNAse) 51.2 1e-05

CAA70141 *Lactobacillus plantarum*; putative protein 51.2 1e-05

YP_396224 *Lactobacillus sakei* subsp. *sakei* 23K; Putative DNA-binding protein, PemK family 51.2 2e-05

YP_794699 *Lactobacillus brevis* ATCC 367; TAT addiction module toxin comp. MazF (an endoRNAse) 50.8 2e-05

ZP_01140449 *Geobacter uraniumreducens* Rf4; transcriptional regulator, PemK family 49.7 4e-05

NP_784302 *Lactobacillus plantarum* WCFS1; HP lp_0524 49.3 5e-05

YP_430997 *Moorella thermoacetica* ATCC 39073; transcriptional modulator of MazE/toxin, MazF 49.3 5e-05

ZP_01188934 *Halothermothrix orenii* H 168; PemK-like protein 49.3 5e-05

YP_359570 *Carboxydothermus hydrogenoformans* Z-2901; regulatory protein, PemK family 48.1 1e-04

GAA02666 *Pelotomaculum thermopropionicum* SI; unnamed protein product 48.1 1e-04

NP_814592 *Enterococcus faecalis* V583; transcriptional regulator, PemK family 47.8 1e-04

NP_470228 *Listeria innocua* Clip11262; HP lin0887 47.8 2e-04

YP_754548 *Syntrophomonas wolfei* subsp. *wolfei* str. Goettingen; transcriptional modulator of MazE/toxin, MazF 47.8 2e-04

ZP_01370257 *Desulfitobacterium hafniense* DCB-2; transcriptional modulator of MazE/toxin, MazF 47.4 2e-04

YP_849071 *Listeria welshimeri* serovar 6b str. SLCC5334; PemK family transcriptional regulator 47.4 2e-04

ZP_01163684 *Lactobacillus reuteri* JCM 1112; conserved HP 47.4 2e-04

YP_076761 *Symbiobacterium thermophilum* IAM 14863; growth inhibitor 47.4 2e-04

ZP_00229889 *Listeria monocytogenes* str. 4b H7858; transcriptional regulator, PemK family 47.0 2e-04

ZP_01149292 *Desulfotomaculum reducens* MI-1; growth inhibitor 47.0 3e-04

YP_877349 *Clostridium novyi* NT; pemK family of DNA-binding proteins 47.0 3e-04

YP_600700 *Streptococcus pyogenes* MGAS2096; MazF protein 46.6 3e-04

ZP_01229280 *Clostridium difficile* QCD-32g58; HP CdifQ_02003809 46.6 3e-04

CAB12273 *Bacillus subtilis*; 1NE8 A Chain A,Ydce Protein 46.6 3e-04

ZP_01273634 *Lactobacillus reuteri* 100-23; PemK-like protein 46.6 4e-04

YP_077792 *Bacillus licheniformis* ATCC 14580; putative plasmid maintenance toxin protein 46.2 4e-04

NP_464414 *Listeria monocytogenes* EGD-e; HP lmo0888 46.2 4e-04

ZP_00740711 *Bacillus thuringiensis* serovar *israelensis* ATCC 35646; MazF protein 46.2 4e-04

ZP_01172374 *Bacillus* sp. NRRL B-14911; HP B14911_20010 46.2 5e-04

NP_830134 *Bacillus cereus* ATCC 14579; PEMK-like protein 46.2 5e-04

ZP_00801948 *Alkaliphilus metalliredigenes* QYMF; PemK-like protein 45.8 6e-04

ZP_01362774 *Clostridium* sp. OhILAs; growth inhibitor 45.8 6e-04

NP_347134 *Clostridium acetobutylicum* ATCC 824; PemK family of DNA-binding proteins 45.8 6e-04

NP_388347 *Bacillus subtilis* subsp. *subtilis* str. 168; HP BSU04660 45.4 7e-04

YP_146086 *Geobacillus kaustophilus* HTA426; HP GK0233 45.4 7e-04

YP_805020 *Pediococcus pentosaceus* ATCC 25745; TAT addiction module toxin comp. MazF (an endoRNAse) 45.4 8e-04

NP_623721 *Thermoanaerobacter tengcongensis* MB4; Growth inhibitor 45.1 0.001

YP_516244 *Desulfitobacterium hafniense* Y51; HP DSY0011 45.1 0.001

YP_174309 *Bacillus clausii* KSM-K16; growth inhibitor 44.7 0.001

ABA71736 *Enterococcus faecalis*; pemK-like protein 44.7 0.001

ZP_00909364 *Clostridium beijerincki* NCIMB 8052; conserved HP 44.7 0.001

YP_535256 *Lactobacillus salivarius* subsp. *salivarius* UCC118; Transcriptional regulator, PemK family 44.7 0.001

YP_697615 *Clostridium perfringens* SM101; PemK family protein 44.7 0.001

ZP_01389611 *Geobacter* sp. FRC-32; PemK-like protein 44.3 0.002

ZP_00778873 *Thermoanaerobacter ethanolicus* ATCC 33223; PemK-like protein 44.3 0.002

NP_561211 *Clostridium perfringens* str. 13; HP CPE0295 44.3 0.002

NP_241388 *Bacillus halodurans* C-125; HP BH0522 43.9 0.002

CAA71064 *Staphylococcus aureus* subsp. *aureus* COL; unnamed protein product 43.9 0.002

YP_819271 *Leuconostoc m*. subsp. *mesenteroides* ATCC 8293; TAT addiction module toxin comp. MazF 43.1 0.003

NP_691544 *Oceanobacillus iheyensis* HTE831; HP OB0623 43.1 0.003

YP_417412 *Staphylococcus aureus* RF122; HP SAB1953c 43.1 0.004

NP_372592 *Staphylococcus aureus* subsp. *aureus* Mu50; similar to pemK family of DNA-binding proteins 43.1 0.004

NP_765227 *Staphylococcus epidermidis* ATCC 12228; HP SE1672 43.1 0.004

YP_300899 *Staphylococcus saprophyticus* subsp. *saprophyticus* ATCC 15305; HP SSP0809 43.1 0.004

ZP_00510624 *Clostridium thermocellum* ATCC 27405; PemK-like protein 43.1 0.004

YP_041518 *Staphylococcus aureus* subsp. *aureus* MRSA252; HP SAR2156 43.1 0.004

ZP_00238203 *Bacillus cereus* G9241; PEMK-like protein 42.7 0.004

AAG23809 *Staphylococcus epidermidis*; AF274004_1 unknown 42.7 0.005

ZP_00540594 *Exiguobacterium sibiricum* 255-15; PemK-like protein 42.7 0.006

YP_252878 *Staphylococcus haemolyticus* JCSC1435; PemK family of DNA-binding protein 42.0 0.008

ZP_01353008 *Clostridium phytofermentans* ISDg; PemK-like protein 42.0 0.009

YP_811155 *Oenococcus oeni* PSU-1; TAT addiction module toxin component MazF (an endoRNAse) 41.6 0.011

ABB17548 *Enterococcus faecalis*; ORFG2-27 41.6 0.012

ZP_01543842 *Oenococcus oeni* ATCC BAA-1163; growth inhibitor 41.6 0.012

ZP_00886168 *Caldicellulosiruptor saccharolyticus* DSM 8903; HP CsacDRAFT_0461 40.4 0.022

ZP_01358153 *Roseiflexus* sp. RS-1; PemK-like protein 40.4 0.024

CAJ71619 *Candidatus Kuenenia stuttgartiensis*; similar to YdcE protein, chain A 40.0 0.035

NP_972847 *Treponema denticola* ATCC 35405; PemK family protein 40.0 0.036

* HP = hypothetical protein; TAT = toxin-antitoxin.
