# Supplementary material for: Plasmids and Rickettsial Evolution: Insight from Rickettsia felis
Source: PLoS One. 2007 Mar 7;2(3):e266. doi: 10.1371/journal.pone.0000266 (PMC1800911; doi:10.1371/journal.pone.0000266)
Supplement: Table S4 — Results of a BlastP search using RP693 (0.04 MB DOC) [file pone.0000266.s004.doc]

**Table S4.** Results of a BlastP search using RP693 (RimJ) as a query. Only sequences with a score greater than 80 bits are shown.

**Accession no. Taxon/annotation* score E**

**(bits) value**

NP_221054.1 *Rickettsia prowazekii* str. Madrid E; ribosomal-protein-alanine cetyltransferase (rimJ) 373 2e-102

YP_067630.1 *Rickettsia typhi* str. Wilmington; ribosomal-protein-alanine N-acetyltransferase 355 8e-97

ZP_00340689.1 *Rickettsia akari* str. Hartford; COG1670: Acetyltransferases, N-acetylases of ribosomal proteins 310 2e-83

ZP_01347904.1 *Rickettsia canadensis* str. McKiel; HP RcanM_01000877 306 3e-82

YP_246234.1 *Rickettsia felis* URRWXCal2; Ribosomal-protein-alanine acetyltransferase 305 6e-82

ZP_00142794.1 *Rickettsia sibirica* 246; ribosomal-protein-alanine acetyltransferase 305 8e-82

NP_360706.1 *Rickettsia conorii* str. Malish 7; ribosomal-protein-alanine acetyltransferase EC:2.3.1.128 304 1e-81

ZP_00154043.1 *Rickettsia rickettsii*; COG1670: Acetyltransferases, incl. N-acetylases of ribosomal proteins 302 4e-81

YP_537668.1 *Rickettsia bellii* RML369-C; Ribosomal-protein-alanine acetyltransferase 283 2e-75

NP_819539.1 *Coxiella burnetii* RSA 493; acetyltransferase, GNAT family 113 5e-24

ZP_01181419.1 *Bacillus cereus* subsp. *cytotoxis* NVH 391-98; GCN5-related N-acetyltransferase 107 2e-22

ZP_01171733.1 *Bacillus* sp. NRRL B-14911; ribosomal-protein-alanine N-acetyltransferase 106 5e-22

NP_241565.1 *Bacillus halodurans* C-125; ribosomal-protein-alanine N-acetyltransferase 105 7e-22

NP_902008.1 *Chromobacterium violaceum* ATCC 12472; prob. ribosomal-protein-alanine N-acetyltransferase 105 8e-22

ZP_00239293.1 *Bacillus cereus* G9241; acetyltransferase, GNAT family 103 3e-21

YP_895482.1 *Bacillus thuringiensis* str. Al Hakam; ribosomal-protein-alanine acetyltransferase 102 1e-20

YP_084316.1 *Bacillus cereus* E33L; ribosomal-protein-alanine acetyltransferase 102 1e-20

NP_845342.1 *Bacillus anthracis* str. Ames; acetyltransferase, GNAT family 102 1e-20

ZP_00393241.1 *Bacillus anthracis* str. A2012; COG1670: Acetyltransferases, incl. N-acetylases of ribosomal proteins 102 1e-20

YP_037073.1 *Bacillus thuringiensis* serovar *konkukian* str. 97-27; ribosomal-protein-alanine acetyltransferase 101 1e-20

NP_832738.1 *Bacillus cereus* ATCC 14579; Ribosomal-protein-alanine acetyltransferase 100 2e-20

NP_979349.1 *Bacillus cereus* ATCC 10987; acetyltransferase, GNAT family 100 3e-20

ZP_01536051.1 *Serratia proteamaculans* 568; GCN5-related N-acetyltransferase 95.1 1e-18

NP_389735.1 *Bacillus subtilis* subsp. subtilis str. 168; HP BSU18530 93.6 4e-18

ZP_00241247.1 *Bacillus cereus* G9241; acetyltransferase, GNAT family, putative 90.9 2e-17

ZP_01185675.1 *Bacillus weihenstephanensis* KBAB4; GCN5-related N-acetyltransferase 90.5 3e-17

ZP_01187625.1 *Bacillus weihenstephanensis* KBAB4; GCN5-related N-acetyltransferase 90.1 4e-17

NP_978761.1 *Bacillus cereus* ATCC 10987; acetyltransferase, GNAT family 89.7 6e-17

ZP_01169229.1 *Bacillus* sp. NRRL B-14911; ribosomal-protein-alanine acetyltransferase 89.4 7e-17

YP_083766.1 *Bacillus cereus* E33L; ribosomal-protein-alanine acetyltransferase 89.0 1e-16

NP_844797.1 *Bacillus anthracis* str. Ames; acetyltransferase, GNAT family 88.6 1e-16

NP_832118.1 *Bacillus cereus*; ATCC 14579Ribosomal-protein-alanine acetyltransferase 88.2 2e-16

NP_831572.1 *Bacillus cereus*; ATCC 14579Ribosomal-protein-alanine acetyltransferase 88.2 2e-16

YP_894486.1 *Bacillus thuringiensis* str. Al Hakam; ribosomal-protein-alanine acetyltransferase 87.8 2e-16

ZP_00236633.1 *Bacillus cereus* G9241; acetyltransferase, GNAT family, putative 87.4 3e-16

ZP_00540263.1 *Exiguobacterium sibiricum* 255-15; GCN5-related N-acetyltransferase 86.7 5e-16

ZP_01173112.1 *Bacillus* sp. NRRL B-14911; Ribosomal-protein-alanine acetyltransferase 86.7 5e-16

NP_978271.1 *Bacillus cereus* ATCC 10987; acetyltransferase, GNAT family 86.3 5e-16

NP_844292.1 *Bacillus anthracis* str. Ames; acetyltransferase, GNAT family 86.3 6e-16

ZP_01254478.1 *Psychroflexus torquis* ATCC 700755; Ribosomal-protein-alanine acetyltransferase 85.9 9e-16

YP_083283.1 *Bacillus cereus* E33L; ribosomal-protein-alanine acetyltransferase 85.5 1e-15

NP_693566.1 *Oceanobacillus iheyensis* HTE831; HP OB2645 85.1 1e-15

ZP_01406156.1 *Acidovorax avenae* subsp. *citrulli* AAC00-1; GCN5-related N-acetyltransferase 84.0 3e-15

ZP_01254495.1 *Psychroflexus torquis* ATCC 700755; Ribosomal-protein-alanine acetyltransferase 84.0 3e-15

ZP_00537937.1 *Exiguobacterium sibiricum* 255-15; GCN5-related N-acetyltransferase 83.2 5e-15

YP_077522.1 *Bacillus licheniformis* ATCC 14580; GCN5-related N-acetyltransferase 83.2 5e-15

YP_724079.1 *Trichodesmium erythraeum* IMS101; GCN5-related N-acetyltransferase 83.2 6e-15

P05332 *Bacillus licheniformis*; YP20_BACLI Hypothetical acetyltransferase p20; 82.8 6e-15

YP_015421.1 *Listeria monocytogenes* str. 4b F2365; acetyltransferase, GNAT family 82.8 7e-15

ZP_00230987.1 *Listeria monocytogenes* str. 4b H7858; acetyltransferase, GNAT family 82.8 7e-15

EAV66955.1 *Burkholderia multivorans* ATCC 17616; GCN5-related N-acetyltransferase 82.0 1e-14

YP_850970.1 *Listeria welshimeri* serovar 6b str. SLCC5334; acetyltransferase, GNAT family 82.0 1e-14

XP_307363.2 *Anopheles gambiae* str. PEST; ENSANGP00000002006 82.0 1e-14

NP_466366.1 *Listeria monocytogenes* EGD-e; HP lmo2844 81.3 2e-14

ZP_00234791.1 *Listeria monocytogenes* str. 1/2a F6854; acetyltransferase, GNAT family 81.3 2e-14

ZP_01183683.1 *Bacillus weihenstephanensis* KBAB4; GCN5-related N-acetyltransferase 81.3 2e-14

ZP_01243090.1 *Flavobacterium johnsoniae* UW101; GCN5-related N-acetyltransferase 80.9 3e-14

NP_623147.1 *Thermoanaerobacter tengcongensis* MB4; Acetyltransferase, incl. N-acetylase of ribosomal proteins 80.5 3e-14

* HP = hypothetical protein.
