# Supplementary material for: Plasmids and Rickettsial Evolution: Insight from Rickettsia felis
Source: PLoS One. 2007 Mar 7;2(3):e266. doi: 10.1371/journal.pone.0000266 (PMC1800911; doi:10.1371/journal.pone.0000266)
Supplement: Table S5 — Comparative genomic analysis of five pathogenetic bacteria harboring plasmids with associated virulence factors. (0.07 MB DOC) [file pone.0000266.s005.doc]

**Table S5.** Comparative genomic analysis of five pathogenetic bacteria harboring plasmids with associated virulence factors.

**Taxon Genome % Plasmid(s) Plasmid % ORFs RNAs**

**size (MB) GC size (MB) GC**

*Bacillus*

*B*. *anthracis* str. "Ames

Ancestor" 5.5 35.4% 2, pXO2 0.09483 33.0% 104 0

pXO1 0.181677 32.5% 204 0

*B*. *anthracis* str. Ames 5.23 35.4% 0 --- --- --- ---

*B*. *anthracis* str. Sterne 5.23 35.4% 0 --- --- --- ---

*B*. *cereus* ATCC 10987 5.43 35.5% 1, pBc10987 0.208369 33.4% 241 0

*B*. *cereus* ATCC 14579 5.43 35.3% 1, pBClin15 0.015274 38.0% 21 0

*B*. *cereus* E33L 5.84 35.1% 5, pZK467 0.46637 33.1% 430 0

pZK5 0.005108 30.9% 5 0

pZK54 0.053501 31.9% 54 0

pZK8 0.008191 31.9% 8 0

pZK9 0.00915 31.0% 10 0

*B*. *thuringiensis* serovar

konkukian str. 97-27 5.31 35.4% 1, pBT9727 0.077112 32.6% 80 0

*Legionella*

*L*. *pneumophila* str. Lens 3.41 38.4% 1, pLPL 0.059832 38.4% 56 0

*L*. *pneumophila* str. Paris 3.64 38.3% 1, pLPP 0.131885 37.4% 139 0

*L*. *pneumophila* subsp.

*pneumophila* str.

Philadelphia 1 3.40 38.3% 0 --- --- --- ---

*Neisseria*

*N*. *gonorrhoeae* FA 1090 2.15 52.7% 0^1^ --- --- --- ---

*N*. *meningitidis* MC58 2.27 51.5% 0^1^ --- --- --- ---

*N*. *meningitidis* Z2491 2.18 51.8% 0^1^ --- --- --- ---

*Pseudomonas*

*P*. *aeruginosa* PAO1 B 6.30 66.6% 0 --- --- --- ---

*P*. *aeruginosa* UCBPP-PA14 6.54 66.3% 0 --- --- --- ---

*P*. *entomophila* L48 5.89 64.2% 0 --- --- --- ---

*P*. *fluorescens* Pf-5 7.07 63.3% 0 --- --- --- ---

*P*. *fluorescens* PfO-1 6.44 60.5% 0 --- --- --- ---

*P*. *putida* KT2440 6.18 61.5% 0 --- --- --- ---

*P*. *syringae* pv. syringae

pv. phaseolicola 1448A 6.11 57.9 2, plasmid large 0.13195 54.1% 127 0

plasmid small 0.051711 56.0% 60 0

*P*. *syringae* pv. syringae

B728a 6.09 59.2 0 --- --- --- ---

*P*. *syringae* pv. tomato

DC3000 6.54 58.3 2, pDC3000B 0.067473 56.2% 70 0

pDC3000A 0.073661 55.1% 68 0

*Yersinia*

*Y*. *pestis* Antiqua 4.88 47.7 3, pMT 0.096471 50.2% 99 0

pPCP 0.010777 45.4% 9 0

pCD 0.070299 44.8% 89 0

*Y*. *pestis* CO92 4.83 47.6 3, pCD1 0.070305 44.8% 71 1

pPCP1 0.009612 45.3% 9 0

pMT1 0.09621 50.2% 101 0

*Y*. *pestis* KIM 4.7 47.7 1, pMT-1 0.10099 50.2% 116 0

*Y*. *pestis* Nepal516 4.65 47.6 2, pMT 0.100918 50.2% 104 0

pPCP 0.010778 45.4% 9 0

*Y*. *pestis* biovar Microtus

str. 91001 4.8 47.7 4, pCD1 0.070159 44.8% 85 1

pCRY 0.021742 49.1% 30 0

pMT1 0.106642 50.3% 122 0

pPCP1 0.009609 45.3% 10 0

^1^ Extreme plasticity and diversity occurs within neisseriae regarding plasmids (van Passel et al., 2006b).
